# Formal and informal science-policy interaction during public health emergencies of international concern: a scoping review

**DOI:** 10.1186/s44263-026-00325-3

**Published:** 2026-09-16

**Authors:** Sophie Meyer, Harvy Joy Liwanag, Kris Schürch, Beatrice Minder, Per von Groote, Caroline Schlaufer, Annika Frahsa

**Affiliations:** 1 Institute of Social and Preventive Medicine, University of Bern, Bern, Switzerland; 2 Graduate School for Health Sciences, University of Bern, Bern, Switzerland; 3 NUS Saw Swee Hock School of Public Health, National University of Singapore, Singapore, Singapore; 4 Public Health & Primary Care Library, University Library of Bern, University of Bern, Bern, Switzerland; 5 KPM Center for Public Management, University of Bern, Bern, Switzerland; 6 Multidisciplinary Center for Infectious Diseases, University of Bern, Bern, Switzerland

**Keywords:** Science-policy interaction, Scientific advisory committees, Public health emergencies

## Abstract

**Background:**

Interaction between science and policy might be a crucial asset for policy decisions during public health emergencies. Much work has been published about processes and forms of science-policy interaction on the one hand and challenges of public health emergency contexts on the other. However, more knowledge is needed to understand the challenges that arise when these two issues intersect.

**Methods:**

We conducted a scoping review informed by the Johanna-Briggs-Institute protocol and searched seven databases to identify formal and informal approaches to organising science-policy interaction. The original search was conducted in April 2022 and updated in March 2026. The searches included Embase.com, Medline, the Cochrane Library, Web of Science Social Science Citation Index, ERIC, Global Health, and Google Scholar. Systematic reviews, articles related to endemic diseases, on clinical and hospital contexts, and impact evaluations of research evidence in decision-making processes were excluded. Information on defined procedures for science policy interaction, form of interaction, selection process of interaction participants, perception of interaction, and barriers and facilitators of the interaction was extracted. Inclusion criteria were a) describe interaction/s of researcher/s and policymaker/s, and b) describe the study setting of a public health emergency. Data analysis followed qualitative content analysis.

**Results:**

87 relevant studies were identified. Most included studies focused on the COVID-19 pandemic, with Scientific Advisory Committees (hereafter SACs) as the dominant mode of interaction. Informal mechanisms, such as committees without formal remit and unclear or undisclosed practices, were less frequently reported but played key roles. Common challenges included unclear roles, political interference, and limited communication. Key requirements are role transparency, formalisation, and institutionalisation of science-policy interaction.

**Conclusions:**

Science-policy interactions during public health emergencies are shaped by dominant formal structures and underexplored informal practices. Strengthening inclusive, transparent, and interdisciplinary advisory systems is key to improving preparedness for future crises.

## Background

Public health emergencies time and again unveil the complexities and challenges of integrating scientific advice into political decision-making. Public health emergencies are characterized by uncertain facts, diverse stakeholder values, significant consequences, and the necessity for rapid decision-making [1–3]. The corona virus disease (hereafter COVID-19) pandemic highlighted the importance of scientific advice to informing political decision-making as indicated by different governments’ claims to be ‘following the science’ to protect their countries from the impact of COVID-19 [4].

Even outside of emergency contexts, translating scientific evidence into policy is a complex process that requires diverse efforts to develop optimal policy solutions, including the synthesis, interpretation, translation, and mobilisation of potentially vast bodies of scientific evidence. To enhance the uptake of research in decision-making processes and, thus policies, knowledge must be tailored to its intended users, i.e. policymakers [5]. Depending on the specific cultural and institutional context, in non-emergency times a combination of conceptual approaches is in place to secure science-policy interaction [6]. In the context of public health approaches of knowledge exchange has been discussed in multiple organisational forms, ranging from linear push or pull process models, in which knowledge is either pushed by producers into the policymaking arena or pulled by policymakers seeking advice, to iterative approaches and coproduction [7–11].

However, public health emergencies simultaneously compress and intensify such demands. Public health emergencies demand immediate action while carrying potentially long-term health consequences at a global level [12]: for instance, worsened mental health outcomes or educational inequalities for children and adolescents [13, 14] or delays in screening, diagnosis, and treatment of diseases [15]. In doing so, public health emergencies “*speed up and challenge the ideal processes of evidence-based policy and practice such that evidence, translation, decision-making, and intervention implementation proceed simultaneously, in greatly compressed timeframes, and in situations with complex intersecting social, economic and political pressure*” [16].

Given the surge in human infectious disease outbreaks in recent decades [17], infectious disease pandemics constitute an interesting and relevant study context for science-policy interaction. They differ in several aspects from other emergencies such as environmental disasters (e.g., bushfires or floods): disease outbreaks occur in wave patterns with possible reinfections, can cross geographic boundaries, are population discriminative, are mutative and adaptive, have a deterministic effect on (health) workforce, and create moving hotspots [18]. For the purpose of this scoping review, we define science-policy interaction as the bidirectional exchange of knowledge, expertise and information between scientific actors and policy actors (e.g., decision-makers like government officials, public health administrations). Such interactions happen at the science-policy interfaces which are “*social processes which encompass relations between scientists and other actors in the policy process, and which allow for exchanges, co-evolution, and joint construction of knowledge with the aim of enriching decision-making*” [19].

So far, research has tended to focus either on processes and forms of science-policy interaction or challenges for science-policy interaction posed by a public health emergency [20–23]. Recently, a scoping review by Bouchat et al [24] integrated both perspectives and examined how policy advisory bodies operated in Europe during the COVID-19 crisis. Their review explored the composition, institutionalisation, formation, and evolution of advisory bodies themselves as well as advisory processes [24]. With this scoping review, we aim to extend the existing insights by broadening the scope both public health-related and geographically: from COVID-19 to further public health emergencies and from a review on literature in the European context to an internationally focused review. We also funnel the scope by focusing our search on how science-policy-interaction is reported upon and discussed in a public health research context.

Interactions of science and policy can be based on formal and informal rules. For this review, we use a working definition of formality as based on set processes and committees with a governmental remit. Conversely, informality refers to committees without formal remit and unclear or undisclosed practices. Our working definition leans on Helmke and Levitsky’s definition of formal institutions as “*rules and procedures that are created, communicated, and enforced through channels widely accepted as official*” and informal institutions as “*socially shared rules, usually unwritten, that are created, communicated, and enforced outside of officially sanctioned channels*” [25]. Still, we recognise the discourse on tricky concepts of formality and informality in political science literature (for differentiated discussion of informal and formal institutions see [25, 26]). This distinction between formal and informal SACs is especially useful in the public health emergency of international concern (hereafter PHEIC) context [27]. PHEICs demand emergency decision-making that combines urgent uncertainty with legitimacy demands, making both institutional forms of advice and its degree of transparency consequential for how responses are produced and justified.

In this review, we therefore focus on science-policy interaction during public health emergencies as a single analytical object, while examining both its function relating to formal and informal establishing procedures and perceived challenges and key requirements from the science-policy interaction in public health emergencies described in the literature. We explore how science-policy interactions are described in peer-reviewed literature on public health emergencies, with a focus on infectious disease outbreaks designated as PHEICs. In this scoping review, we focus on the function of science-policy interaction in public health emergencies relating to formal and informal establishing procedures and perceived challenges and key requirements from the science-policy interaction in public health emergencies described in the literature.

## Methods

**Fig. 1:**
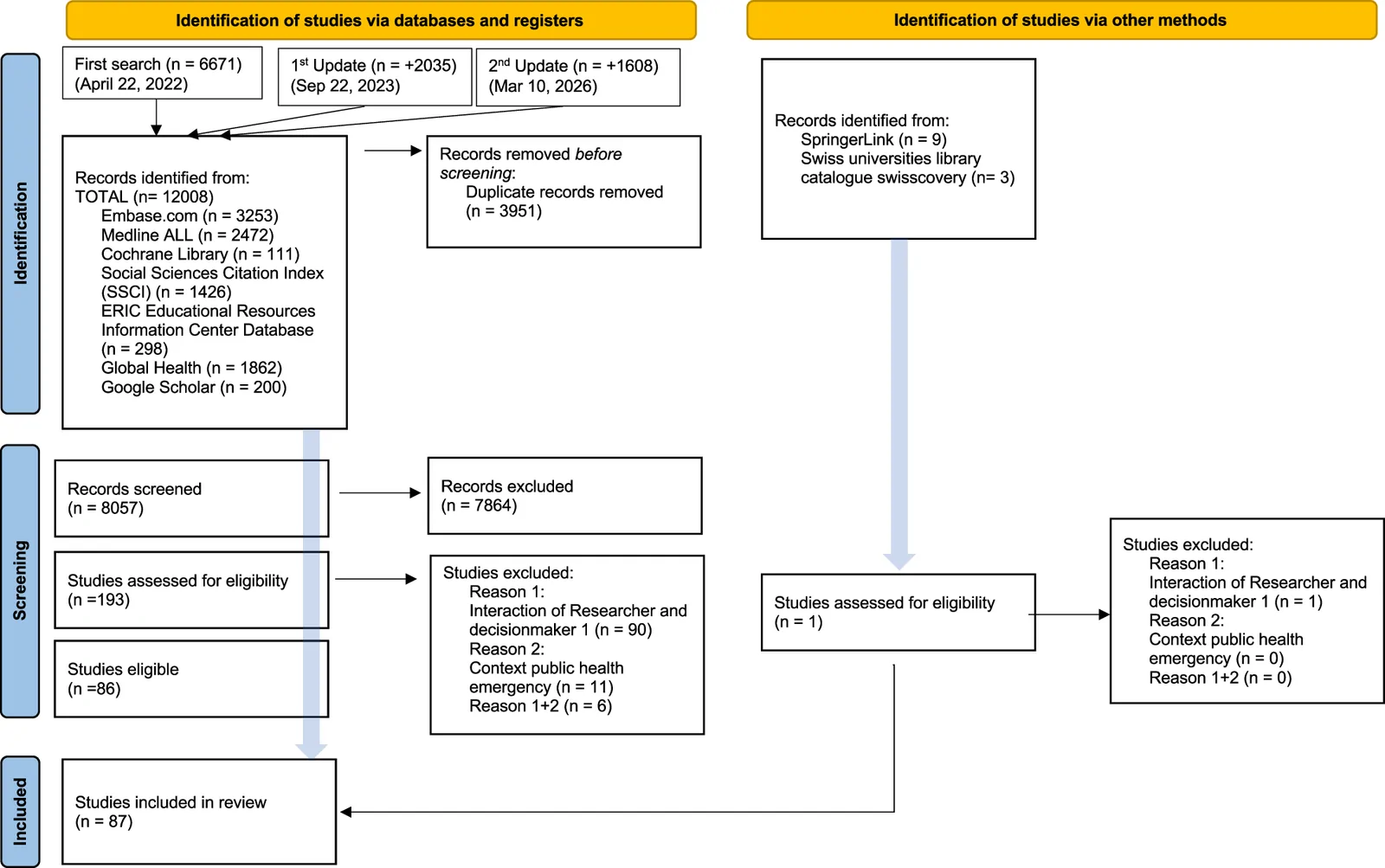
PRISMA flow diagram for scoping reviews

Scoping reviews “*systematically map the literature available on a topic, identifying key concepts, theories, sources of evidence and gaps in the research*” [28]. In this scoping review, we followed the JBI guidance for conducting scoping reviews [29]. The reporting of the scoping review (Fig. 1) adhered to the PRISMA extension for scoping reviews (PRISMA-ScR) [30]. and yielded 6461 articles to screen in title and abstract. The PRISMA checklist can be found in Supplementary Material 1: PRISMA-ScR) Checklist. The study protocol was registered on the Open Science Framework in June 2022 [31]. This review was conducted collaboratively with researchers from various disciplines (SM, HL, KS, PvG, CS, AF) and supported by one medical information specialist (BM) who developed the search strategies and conducted the searches.

### Search strategy and sources of evidence

We used a combination of search terms related to four main areas: (1) decision making, health care policy, crisis management, (2) knowledge translation, evidence (scientific, research-based), (3) public health emergencies, and (4) associated approaches, tools, guidelines, frameworks. These terms were initially based on [12] and were expanded through expert input. Because scientific policy advice can take many forms and routes, we searched both for individuals (e.g., expert or broker) and groups (e.g., committee or expert meeting). The systematic searches included Embase.com, Medline (Ovid), the Cochrane Library, Web of Science Social Science Citation Index (SSCI), ERIC (Ovid), Global Health (Ovid), and Google Scholar.

We complemented these with hand searches of the Swiss universities’ library catalogue (swisscovery) and SpringerLink for monographs and book chapters, and we asked experts in the field, recruited through the author’s professional networks, to suggest additional key publications, including monographs and book chapters. Our search strategy encompassed peer-reviewed literature (including case studies, review articles, and commentaries) with no language or time restrictions applied. The search was originally conducted in April 2022 and was updated on 22 September 2023 and again on 10 March 2026. The complete search strategies are provided in Supplementary Material 2: Full Search Strategy per Database.

### Eligibility criteria

In screening title and abstracts of 8057 articles, we excluded systematic reviews and articles related to endemic diseases such as malaria, dengue, chikungunya, and cholera, since these infectious diseases tend to be part of longer-term public health and health systems decision-making processes. Due to the review’s focus on science and policy interaction during emergencies, we excluded articles on clinical and hospital contexts (e.g., patient medical decision making), articles reporting about prevention (even if part of an infectious disease like human immunodeficiency virus), as well as impact evaluations of research evidence in decision-making processes. To be included for full text screening, articles needed to cover the following criteria: a) describe interaction/s of researcher/s and policymaker/s, and b) describe study setting of a public health emergency. We included peer-reviewed original articles, commentaries and perspectives articles, as well as book chapters. For empirical study types, we focused on both quantitative and qualitative research, but excluded randomised controlled trials given our interest in research about real-world applications of science-policy-interaction.

### Screening process

We collated and uploaded all identified articles into EndNote 20.1 (Clarivate Analytics, PA, USA) and removed duplicates using Deduklick [32].We conducted title-abstract-screening in EndNote20, following [33]. We entered all data directly in the web-based database Research Electronic Data Capture (REDCap) platform hosted by the University of Bern. We screened in two steps: (a) title and abstract, and (2) full text. In step one, we screened in duplicate by four researchers and the team met to discuss discrepancies in inclusion or exclusion decisions. We applied the same procedure in full-text screening to ensure all relevant studies were captured before proceeding to data extraction.

### Data extraction charting and analysis

**Fig. 2:**
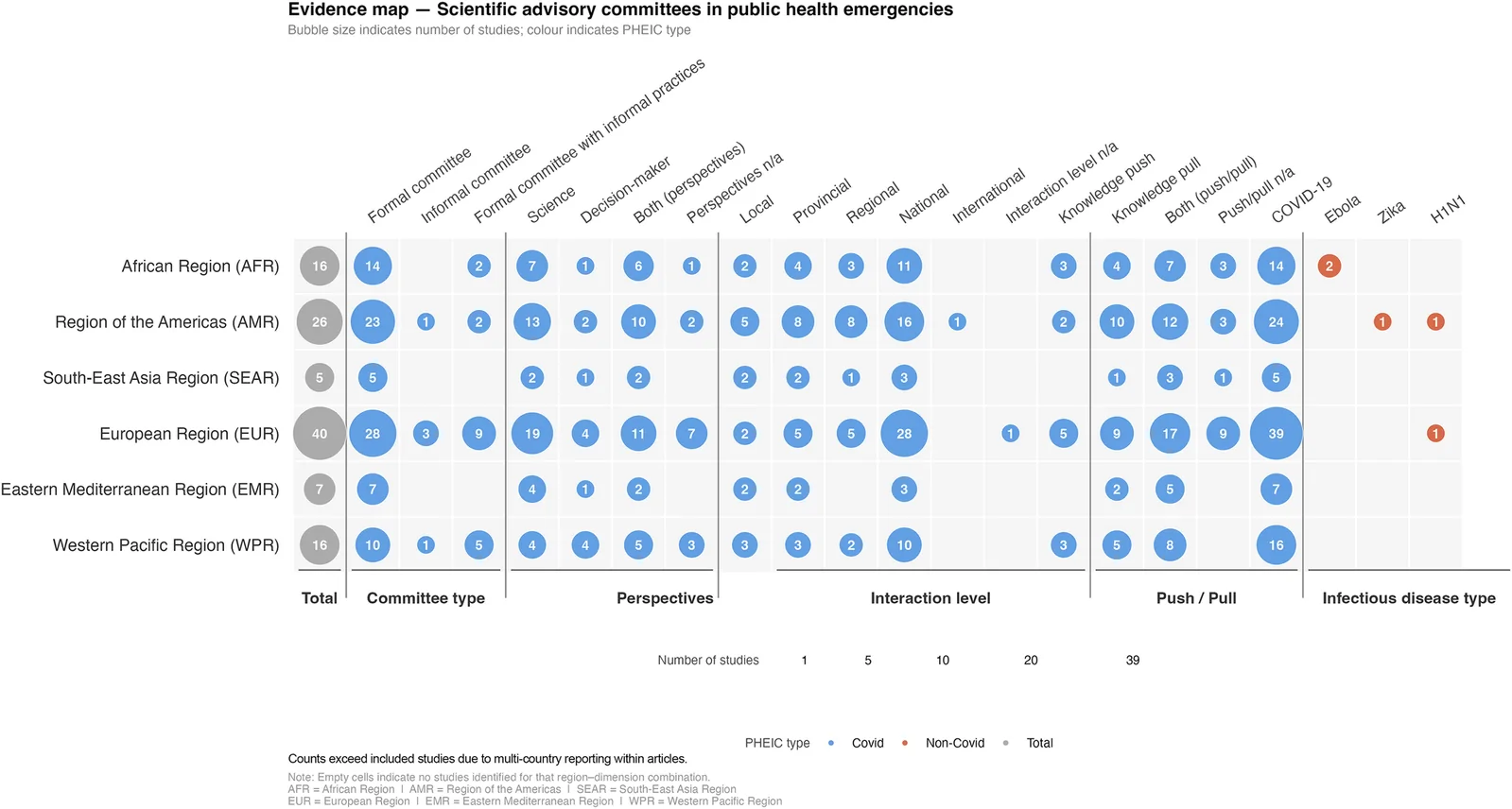
Evidence map of included articles

The research team created and pilot-tested the data extraction form, and the lead author completed data extraction from the articles, discussing difficulties as necessary with co-authors. Among the details that we extracted are defined procedures for science policy interaction, form of interaction described, selection process of interaction participants, and perception of interaction as well as barriers and facilitators of the interaction. Additionally, we extracted contextual data (e.g., countries and infectious diseases, level of interaction, knowledge push and pull mechanisms). The data extraction form can be found in Supplementary Material 3: Data extraction form. Supported by qualitative analysis software (MAXQDA2024 Analytics Pro 24.110, VERBI Software, Berlin), we conducted qualitative content analysis [34, 35] of the data with both inductive and deductive category building (for overview on thematic categories and subcodes see Supplementary Material 4: Coding framework). Data visualisation (Fig. 2) was created using RStudio (Posit Software, PBC, Boston, MA, USA, 2025.09.2 + 418 using ggplot2 package). As recommended by the methodology [36], the research team met to discuss and refine data extraction criteria in an iterative and reflexive process over the course of the project timeline.

## Results

In total, there were 8057 articles to screen in title and abstract. Although we were interested in various forms of science-policy interaction, including researchers’ interactions with decision-makers at individual levels, all but one of the articles identified from our search portrayed collective forms of interaction, mostly through SACs. This section presents the findings from the 87 included studies in three stages. First, we provide a descriptive overview in drawing the landscape of literature including geographic distribution, types of PHEICs addressed, as well as temporal, theoretical, and jurisdictional patterns. Second, we examine the literature through the analytical distinction between formal and informal SACs, presenting key characteristics and findings within each category. Finally, we synthesise cross-cutting challenges and key requirements that emerge across the entire body of evidence, irrespective of SAC type.

### Landscape of literature on science-policy interaction

#### Country distribution of studies

Among the 87 articles, 73 reported on a single country, and 16 articles reported on or compared two or more countries (for characteristics of included studies, see Supplementary Material 5: Characteristics of included studies). All six world health organisation (WHO) regions were represented in the review. Of 70 countries covered in the articles, 31 were High Income Countries, and 39 were Low- and Middle-Income Countries in accordance with the World Bank’s practice for income classification. In our data corpus, studies focused disproportionately on the United Kingdom (UK) (*n* = 19) and Canada (*n* = 15), indicating an overrepresentation of these countries. Figure 2 shows an evidence map with the respective numbers of included studies relating WHO regions, committee type, perspectives portrayed, interaction level, push and pull mechanisms and PHEICs described.

#### PHEICs described: dominance of COVID-19

While the scope of this review encompassed infectious disease outbreaks designated as PHEICs, our systematic search identified a concentration of literature on the COVID-19 pandemic (82, 95%). Only five included studies addressed non-COVID-19 outbreaks (e.g., Ebola, Zika, H1N1). The dominance of COVID-19 literature in our final sample reflects both the recency and volume of research on science-policy interactions during this pandemic and shapes the empirical grounding of our findings. For characteristics of PHEIC that influenced science-policy interaction, see Supplementary Material 6: Additional insights from extracted data, chapter I. Based on the included articles, we found no significant difference in SAC formation, facilitators or perceived challenges between COVID-19 settings and other PHEICs such as H1N1, Zika, or Ebola. Based on our corpus the below-described challenges and key requirements thus seem to hold true for SAC work in public health emergency settings.

#### Temporal, theoretical, and jurisdictional patterns

Only three articles [37–39] had been published before 2020. One of the 87 articles was a book chapter, while 75 were peer-reviewed original research, 9 were commentaries and 3 were reports. Articles published in or in direct aftermath of the COVID-19 pandemic tended to be either peer-reviewed reports, perspectives, or commentaries presenting anecdotal descriptions of lived events (23, 29%), with few of the articles with an explicitly stated theoretical underpinning or some form of reflexivity. In contrast, articles that were published from 2022 until 2025 increasingly applied social science theories and methods to analyse the interaction itself.

Also, 71 articles presented researcher-policymaker interaction on a national level, 19 articles on regional, 24 articles on provincial, 16 articles on local and one article on an international level. 52 articles described a knowledge exchange, while 31 articles described a knowledge pull, and 13 articles a knowledge push in the interaction.

The included studies examined interactions between researchers and decision-makers at local, provincial, regional, and national levels. Our analysis revealed no significant differences in the challenges or enablers of these interactions based on jurisdictional levels, except in one case in which jurisdictional differences led [40] or were assumed to lead to tensions with an established Scientific Advisory Committee [41].

### Formal committees

#### Established infrastructures

In 40 cases (45%, accounting for a total of 29 countries), formal systems had existed prior to the emergency [38, 40, 42–74]. These can also include adapted pre-existing institutions [50, 75], capabilities and relationships [68, 69, 71, 76, 77]. Several of the pre-existing formal systems were administration-related or administration-internal institutions, such as national public health institutes, which acted as the scientific advisory body [42, 43, 45, 47, 50, 55, 68, 69, 73, 78]. Only one paper described an individual in a scientific advisory role: the chief medical officer depicted as highest-ranking government advisor on health issues working at the intersection of state, medicine, and civil society [72]. Neil-Sztramko et al revealed that Canada presented itself as a peculiar case of a full-grown science advisory ecosystem: shaped by the SARS and H1N1 pandemics, the science advisory landscape of National Collaborating Centres for Public Health acted as “*evidence intermediary”* [61], and the system was further populated by various ad hoc SACs during the COVID-19 pandemic [79].

#### Formation of SACs: governmental and academic initiatives

Half of the articles described the process of establishing SACs, at least to some degree. Governments were perceived as the main driving force in their creation [38, 39, 45, 46, 49, 51, 55, 56, 60, 61, 63–65, 67–69, 73, 75, 78, 80–100]. König and Stampfer reported on the informal committee which in this respect is a unique case, being independent but initiated by the adjutant of the Austrian Armed Forces to the Austrian Federal President and former Minister of Defense [91]. As described above, in several instances, decision-makers chose the members of the SAC, in other cases individual experts proposed themselves [40, 101] or were recommended by professional associations [95, 102] with the final approval made by policymakers. In two cases, an SAC was established by a shared initiative of government and academia [41, 53, 76]. Sometimes the initial effort was made by researchers and subsequently adopted by a governmental mandate [90, 103, 104], in one case the scientific initiative remained without a formal mandate [105]. In one case the establishing effort was made by a not-for-profit organization [106]. Based on formal practices or not, many SACs were established ad hoc during COVID-19 [39, 41, 51, 55, 65–67, 69, 78–80, 82–84, 87–89, 92–97, 100, 102–104, 107] and the swine-flu [37].

#### Frameworks and models guiding science-policy interaction

From the 87 included articles, four described a framework, e.g., knowledge translation platforms or the WHO Strategic Response Framework, pandemic preparedness plans [37, 40], or a model, e.g., the WHO Evidence Informed Policy Network [42] to establish interaction or the WHO-INTEGRATE Evidence-to-Decision framework to develop a public health guideline [73]. Other reported approaches included diverse concepts to inform science-policy interaction, ranging from “*honest knowledge brokering*” [108], participatory approaches, OneHealth approaches, national collaborating centres, learning health systems, to disaster management apparatuses [41, 92, 93, 104, 109].

Additionally, authors of included articles suggested new frameworks and models: to co-produce evidence between political decision-makers, health policymakers and academics’ [80], to improve decision-making in the face of epistemic and strategic uncertainty, and institutional uncertainty based on their six-country empirical research [69], to guide the use of modelling data for policy decision-making, building on insights derived from their findings [110], a conceptual framework for effective evolving data ecosystems that demonstrate strong institutional leadership and adapt strategically to meet policymakers’ information requirements [71], and a “*novel form of research translation, co-creation and data collection to employ in a crisis setting*” called Functional Dialogues [76].

#### Networks and neutrality: implications of expert selection

In several cases, selection of SAC members relied on previously established science and policy relationships [37, 40, 41, 50, 54, 57, 67, 69, 71, 78, 81, 84, 88, 89, 96, 106, 110–113]; in one instance, personal ties based on close prior collaboration facilitated the SAC involvement of researchers [51]. The selection of SAC members based on previous collaboration, however, was also reported in relation to one informal committee [78] (For additional findings on composition and omissions in SACs, see Supplementary Material 6: Additional insights from extracted data, chapter II).

Since previous collaboration often determined SAC membership, Williams et al questioned the neutrality and independency of chosen experts [67]. Dostal argued that the German government’s choice of unbalanced and handpicked SAC composition resulted in a very narrow focus or perspective [49].

Formal SACs are typically designed to operate within clearly defined and declared procedural frameworks. However, the literature reviewed in this study revealed that informal practices were deeply embedded within these formal structures.

#### Informal practices in formal SACs

Informal practices took place in formal SACs, for instance, through decision makers’ deliberate selection of a specific person as an SAC member. In several cases, committee members were reported as directly chosen or “*hand picked*” [65, 71] by policymakers [45, 46, 49, 55, 56, 80, 83, 87, 95, 96], in some instances based on their political alignment [98]. Howard and Hyland-Wood described the Australian case of infectious disease modelling, the “*hand picking*” of advisors by the federal government was reported to limit and withhold its modelling assumptions, which hindered independent scrutiny [71]. According to Easton et al political parties in Belgium interfered in membership selections, suggesting “*trusted insiders*” [50]. SAGE members were reported at first to be undisclosed [55, 105] and their selection criteria were either unclear or opaque [62, 63]. Additionally, Jarman et al mentioned that the formal advisory bodies in the UK, France, and Germany were populated by more informal actors like special advisors and consultants [55].

Informality in communication channels also appeared to be a common practice of policymakers working with researchers. Describing evidence use in decision-making during the COVID-19 pandemic in British Columbia (CA), Brubacher et al provided perceptions of both researchers and health officials about unclear pathways of how information ultimately found its way to policymakers: the used channels were depicted as unclear or perceived as “*organic*”. These informal traits eventually led to the perception of a lack of a formal, independent SAC [81]. Similarly, in the UK case, described by Hammersley, communication channels were “*private and hierarchical*” [52]. Easton et al reported about informal contacts between administration and experts in Belgian crisis management, which subsequently led to informal new structures based on these contacts, underlining the considerable influence of informal contacts in policy decisions [50].

### Informal committees

In addition to informal practices within formal structures, the literature also pointed to the existence of SACs that are informal by design. These entities operate outside established institutional frameworks and lack formal mandates. Nevertheless, they play a significant role in science-policy interactions by providing flexible and rapid advice. This chapter examines how such informal committees emerged, how they functioned and how authors perceived their advantages compared to formal SACs.

Five articles described advisory bodies that were informal by definition: the Austrian informal exchange platform [91], the “independent SAGE” in the UK [52, 105], the “*informal group of epidemiologists*” in North Carolina, United States of America (USA) [78], and the independent and informal science advisor OCTA Research Group in the Philippines [107]. While the former three were developed out of researcher’s own initiative and perceived need for an informal scientific advisory committee, the latter was created due to policymaker’s demand but without formal mandate. Informal committees may come with benefits. König and Stampfer argued that the Austrian informal exchange platform was presented with advantages in comparison to the formal SAC bodies: it provided a space for policy innovation without political interference, maintaining independence in contrast to formal bodies that struggled to separate facts from interests and short-term actions from long-term strategies [91]. It aimed to provide policymakers with evidence for decision-making, but struggled too due to policymaker’s reliance on informal, reactive communication channels [91]. Still, although Weinkle et al described advantages of an SAC without formal remit as flexible and “*quick in production of perceived policy relevant advice*” [78], they acknowledged the necessity of a standing SAC.

### Synthesis of challenges and key requirements

Many of the included studies provided information on challenges, reflections on the double-edged role of experts, and key requirements from these interactions. Regardless of the format of engagement being formal or informal, this section synthesizes these cross-cutting insights, offering a thematic overview of how these experiences were appraised in the included literature. Challenges in the science-policy interaction can arise from diverse mechanisms.

#### Missing exchange mechanisms

Missing institutional mechanisms for science-policy exchange posed a challenge for quick and effective collaboration [102, 114], for instance lacking structures and processes to efficiently handle information and make effective decisions [62]. Conversely, Tuohy et al reported upon the UK context that although an established path can reduce transaction costs it may also create costs when it becomes necessary to leave said path, for example in an unprecedented situation like the COVID-19 pandemic [100].

#### Knowledge translation and usability

Many challenges reported by the articles were communication-related. Articles reported difficult science-policy communication that was mostly related to the lack of governmental understanding of science and scientific methods [49, 66, 77, 82, 100, 115, 116], especially regarding scientific uncertainty [37, 103, 117]. Corrin et al highlighted that overly technical evidence could overwhelm decision-makers, leading to misinterpretation or disregard [77]. Questions by policymakers were formulated in an unanswerable way by synthesis requestors, which in turn led to frustrations by knowledge producers in Canada [77]. However, researchers too lacked knowledge of political processes, leading to politicians criticising SACs for failing to function as they should, namely “*at the service of politics and not vice versa*” [89].

#### Internal and external governance of SAC

Within SACs, missing consensus methods [63] and an inability or challenge to define a clear message due to an exceedingly large expert group [39, 118] were noted as further challenges. Other internal challenges related competing interests of stakeholders [84, 97] and rivalry between ministries [65].

#### Political filtering and selective uptake

Challenges in science-policy interface can also relate to it being shaped by power. Abubakar et al saw a challenge for SAC work when it was governed in a directive nature limiting topics and breadth of subjects that reduced potential useful advice [80]. Corruption and nepotism were cited as challenges that could be potentially bridged by a knowledge translation and exchange strategy [119]. Some articles described the perceived ignorance of scientific advice by governments. Cases stemmed from all over the world, from Latin America [110], the UK [55, 64, 105], the US [68], Sweden [45], and Canada [120]. Occasionally, researchers went to the media when they felt that policymakers were not considering their advice [40, 45, 82, 105, 110, 116].

#### Boundary work and role ambiguity

Researchers perceived challenges also in terms of defining roles and responsibilities [50], such as identifying who qualifies as a public health expert, which became challenging in British Columbia once politicians and bureaucrats entered the sphere of public health expertise [81]. Included articles diverge on the usability of intermediaries: one multi-country study on knowledge translation mechanisms found that both researchers and policymakers preferred face-to-face exchange and disliked intermediaries brokering knowledge [110]. However, de Andrade et al reported that in Brazil, they have played an important role, acting as neutral communicators between the generator and the user of produced knowledge [70].

Several articles charted instances of researchers explicitly overstepping science and policy interaction boundaries by talking directly to the public and media when governments wanted researchers to talk to them exclusively [40, 67, 87, 107]. Camporesi et al highlighted that in Italy, SAC members signed nondisclosure agreements prohibiting contacts with the media [46], Tunis et al reported that the Terms of Reference of the Canadian National Advisory Committee on Immunisation (NACI) were clear in prohibiting members to speak publicly on behalf of the NACI’s independent advice unless asked to do so by the Public Health Agency [74].

Although researchers recommended otherwise, in several instances policymakers were not (always) guided by scientific evidence [64, 65, 73, 82, 91, 92, 102, 111, 115, 119]. As reported by Brusselaers et al in Sweden, several ‘experts warned about the COVID-19 pandemic already in January 2020, but this was dismissed by the authorities and even ridiculed in the media’ [45]. Brusselaers et al even predicted a decline of trust in future cooperation between science and policy due to the COVID-19 pandemic experience.

Role ambiguity may escalate into a substantive blurring of the science-policy boundary, where unclear mandates allow advisory bodies and policy actors to conflate initially separated roles.

#### Blurring boundaries between science and politics

Boundaries between science, policy, and politics can also blur as academic neutrality is given up for the sake of policy [67, 92, 111]. Camporesi et al pointedly reported on SACs quasi-legislative function: the Government requested the SAC minutes in Microsoft Word format instead of PDF to streamline the drafting of decrees [46]. According to Hammersley this direct translation of what measures to be taken based on researchers’ advice was perceived as outside of the competency of science in the UK [52]. Aagard et al described a blurring of technical and political expertise in Denmark, especially in public representation through press conferences [68]. Authors shed light on crucial challenges facing some SACs: scientific independence in danger [59, 66, 79, 87, 88], and general conflicts of interest [97]. Conflation of roles as knowledge producers and knowledge users also can lead to normative judgements or "*downplaying the uncertainty within their own models as they provided advice to decision makers*" [63]. In the special case of the chief medical officer in the UK, independence got challenging once they had to speak on behalf of the government and speak on behalf of science [72]. Another facet to this is the freedom to criticise political decisions which seemed to be limited whilst being member of an SAC [50].

In several instances, evidence was invoked as justification for governmental actions [40, 49, 50, 59, 68, 78, 82, 107, 115, 120] such as closing businesses or holding up municipal elections [65, 94] or to reinforce actions already taken [59]. Oztig mentioned that in Turkey’s case, due to the public reputation of “*doctors and scientists*” as “*trustworthy*”, the “*following the science*” narrative sustained prevailing policy styles such as low-level inclusion in decision‐making and the depoliticization of issues at stake [94]. Christensen et al reported that early in the COVID-19 pandemic in Norway governmental leaders held daily press conferences together with expert leaders to clarify the situation, reassure the public, and promote adherence to guidelines, which bolstered their own legitimacy leaving little room for open disagreement [121]. Articles reported that evidence was used for decision making, but not in the right way by the right institutions at the right time [62].

Out of the reported challenges, authors of included studies distilled practical key requirements for future emergency SAC work.

#### Key requirements for effective and sustainable SACs

Articles mentioned the need to differentiate SACs and governments’ roles and responsibilities [96]. This was highlighted by Waganew et al who argued for avoiding role confusion, foster clear coordination and communication [97], with the latter being also mentioned by Mbachu et al [122]. McSween et al suggested improving communication loops to better align knowledge producer responses with decision-makers’ needs [59].

Others called for the necessity or opportunity [79] to formalise and institutionalise relationships and collaboration for future emergency situations [75, 102, 106, 122, 123], as well as infrastructure investments for stronger and more streamlined collaboration and uptake into policy decisions [81]. Authors described a necessity to ‘establish a standardized mechanism the scientist group to participate in risk decision-making’ [112], to develop “*a structured format for receiving queries will facilitate the process by streamlining the flow of information*” [42], to “*create a standing science advisory committee to serve the governor during times of health crises such as a pandemic*” [78], or to maintain partnerships [77], as trust and relationships “*can’t happen overnight during an emergency*” [110]. As Hadorn et al aptly summarised: “*Established cooperation between experts and politicians makes it easier to refer to these routines in times of acute need for knowledge in times of sudden uncertainty*” [40]. Furthermore, investments in capacity building, data infrastructures and funding schemes were perceived as essential for successful knowledge translation in future scenarios [70, 110]. Corrin et al described enhancing decision-makers’ knowledge about nature and potential usage of evidence, for instance, knowledge synthesis as an ‘area for improvement’ [77]. Additionally, establishing centralized and strong leadership for research during public health emergencies was suggested to help to ensure clear direction, coordinated efforts, and effective collaboration, minimizing fragmentation and duplication [50, 81]. Wabnitz et al perceived a necessity to discuss for future emergency settings, whether and what type of advisory institutional arrangements are necessary to inform public health policy making [73].

The scientific independence of SAC was reported to be essential for SAC work. Brubacher et al described the need to protect the independence of public health (research) to act and speak as a crucial component of good governance [81].

Looking at SAC work from an academic perspective, Hillmer et al and Sherratt et al argued that universities should recognize scholars’ crisis-related contributions beyond teaching and research, integrating such work into promotion and tenure decisions to avoid discouraging future engagement [41, 123].

## Discussion

This scoping review explored how science-policy interactions were described in peer-reviewed literature on public health emergencies, with a focus on infectious disease outbreaks defined as PHEICs. We focused on the function of science-policy interaction in public health emergencies relating to formal and informal establishing procedures and perceived challenges and key requirements from the science-policy interaction in public health emergencies described in the 87 included articles.

Our analysis revealed a marked increase in the number of studies during and following the COVID-19 pandemic. SACs and PHEIC have emerged as prominent topics of interest primarily since the onset of COVID-19. Prior to 2020, we found limited literature addressing these topics in the context of other infectious disease outbreaks.

This strong concentration of studies on the COVID-19 pandemic reflects both the unprecedented scale of this pandemic and the surge in research activity it generated, but also reveals a gap in the literature regarding science-policy dynamics in other types of emergencies. Moreover, although we searched for a range of interactions between science and policy, the review showed that formal advisory structures, particularly SACs, were the dominant form of interaction reported, while informal mechanisms, including ad hoc groups and interpersonal exchanges, were less frequently explored yet played crucial roles. Across both formal and informal settings, challenges such as unclear roles, limited communication channels, and political interference were frequently highlighted. These findings underscore the complex and often contested terrain of science-policy exchange during crises, highlighting opportunities to strengthen preparedness, inclusivity, and responsiveness in future emergency contexts.

Additionally, we observed a geographic concentration on Europe and North America. This concentration likely reflects both the distribution of research production and the indexing of literature in major databases, which may privilege journals and institutions from Europe and North America.

While our review covers a broader geographical and thematic scope, our findings on science-policy interaction during public health emergencies resonate with key insights from Bouchat et al [24], who examined advisory body composition and selection processes during COVID-19 in Europe. Their analysis highlighted direct appointment of SAC members by executive actors, raising concerns around transparency, impartiality, and independence—issues that also emerged in our global dataset. Similarly, although many articles in our review referenced pre-existing formal advisory systems, we observed a consistent absence of clear guidelines or terms of reference for SACs, confirming patterns identified by Bouchat et al. Our findings thus support and broaden their conclusions, demonstrating that these governance challenges are not confined to the European context or to the COVID-19 pandemic alone.

In infectious disease emergencies, science-policy interactions often diverge from routine processes. Urgency, uncertainty, and high stakes increase intensify reliance on informal mechanisms, such as selective SAC appointments and exclusive communication [45, 124]. While committees may be formally constituted and governed by explicit procedures, their day-to-day functioning may reflect enacted informal practices, suggesting that formally described structures do not always correspond to how advice is actually produced and negotiated. Informality can shape policymaking in ways formal rules do not [25], likely influencing science-policy exchange beyond what is captured in the literature. While informal interactions offer speed and flexibility [78], transparent and conflict-free SAC member selection remains essential [125]. Our findings reinforce the literature on the importance of SAC design features such as size, diversity, procedural clarity, and effective communication [57, 126].

Institutionalising SACs presents a dual effect: pre-existing relationships can enhance responsiveness but may also risk nepotism and over-reliance on individual expertise [40]. In crisis contexts, policymakers may turn to epistemic communities with shared worldviews, reinforcing dominant perspectives while marginalizing others [51, 127]. Establishing SAC structures outside emergency settings may help avoid short-termism and preserve institutional memory [62]. Additionally, pre-pandemic preparation and institutionalisation could streamline science-policy interactions and maintain focus on long-term goals amid immediate demands.

The idea that science and policymaking can be seamlessly linked, often termed “*naïve evidence-based policymaking*”, has long been critiqued for ignoring structural mismatches and power asymmetries at the science-policy interface, and instrumental legitimation of evidence [124, 128]. Our findings underscore this perception with several included articles raising concerns about scientific independence and the quasi-legislative roles SACs sometimes assume.

These concerns point to a broader theme: science-policy interaction during PHEs is shaped by its political context. This was evident in our findings, such as the executive appointment of SAC members, the recurrent reports of political interference and the use of scientific advice to legitimate decisions taken on other grounds [124, 128]. Under acute pressure, governments may also rely on trusted individuals or established epistemic communities, reinforcing dominant perspectives while marginalising others [51, 127]. Informal interactions are particularly exposed to these dynamics, as they operate outside the procedural safeguards of formal structures [25]. The frequent concerns about scientific independence and role ambiguity likewise reflect that the boundary between providing evidence and exercising political judgement is itself politically negotiated [108]. Recognising this political character does not diminish the value of scientific advice but underscores that strengthening the interface is as much about managing political relationships as about improving technical processes.

While transdisciplinary and co-production approaches, such as boundary-spanning roles, research-practice partnerships, and knowledge brokers, have shown promise in public health [8–11, 129], our review found limited uptake of such models during emergencies.

Challenges around scientific autonomy and role ambiguity suggest that researchers must reflect critically on their engagement in advisory roles [108]. Thus, clarifying responsibilities and decision-making authority remains essential for effective and accountable science-policy collaboration [66].

The recommendations focus on SACs because they were the most commonly reported and most consistently described form of science-policy interaction in the reviewed literature. This does not imply that other forms are unimportant, but rather that they were less visible in the evidence base retrieved for this review. Based upon the findings from the review, we derived the following recommendations to enhance science-to-policy interactions during Public Health Emergencies, such as PHEICs:

- Election of SAC members: SAC members should be elected through a process that prioritises professional independence and scientific competence, selecting candidates on the basis of disciplinary expertise and track record rather than political alignment or personal connections.
- Institutionalisation, Independence, and Sustainability: Most SACs were established by governments on an “ad hoc” basis despite previous repeated reminders from global health organizations like the WHO to prepare for Public Health Emergencies even before they occur. We recommend the institutionalization of SACs as part of routine pandemic planning where committees are established well before the next Public Health Emergencies and already prepared to respond to Public Health Emergencies in an agile manner. Institutionalisation of SACs will require enabling policies, including policies that protect their independence from political interference and ensure the allocation of resources to support their mandate and activities in a sustainable way.
- Clear and Transparent Process and Accountability: For an effective workflow of SACs, a delineation of the roles of members and staff and transparency in standard operating procedures are needed on what SACs are mandated to deliver and how SAC recommendations are used to inform government decisions and policy development. Clarity of roles and procedures also helps to promote SAC accountability.
- Diverse Composition: The findings from this review reiterate the prevailing dominance of the biomedical and natural sciences in the composition of SACs, which has contributed to a sub-optimal response to Public Health Emergencies. Responding to Public Health Emergencies would also require attention to the social and behavioural dimensions of health. A priori inclusion of a wider range of disciplines will foster a holistic understanding of health dimensions and consider diverse epistemological approaches. In addition to a multidisciplinary composition that includes the behavioural and social sciences, we recommend the SAC composition to be gender-balanced, and multi-stakeholder in perspective, including the voice of people with lived experience. SACs for Public Health Emergencies could learn from the well-established system of research ethics committees that require the participation of community and lay representatives in their discussions and decision-making [130].
- Cross-Country Learning: Our review revealed many SACs in a wide range of contexts, yet no international cross-country learning platform has been established for SACs. We propose the consideration of a cross-country mechanism that could bring SACs between and within countries together to foster mutual learning, networking, and potential benchmarking.

- Awareness: Our review found science-to-policy informal interactions to be less documented and less studied in the literature despite being relevant in impacting the Public Health Emergencies response. It is important to foster better awareness of these informal interactions and to examine how they could be leveraged to complement, rather than compete with, the existing formal interactions.
- Relationship Building: Interactions are also largely based on trust and the pre-established relationships among researchers, policymakers, and other stakeholders in the ecosystem. We therefore recommend also the importance of better understanding how best to create and build upon these relationships in the science-to-policy ecosystem to support a more effective overall response to Public Health Emergencies.

Within the PHEIC-focused literature identified by this review, relatively few studies in-depth examined science-policy interactions in low- and middle-income countries (LMICs) settings, compared to the volume of articles describing detailed experience from high-income countries. We recommend further research to document and analyse science-to-policy interactions during PHEs in LMICs to obtain a more complete global picture of the landscape of the evidence, including identifying contextual and cross-contextual lessons for enhancing interactions, cross country learning and relationship building.

This finding may reflect not only where science-policy interaction occurs, but also how it has been operationalised in the literature we identified, which centred largely on formal advisory structures such as SACs. These represent one organisational form among several, and evidence from LMIC settings suggests that comparable functions are frequently carried out through other arrangements, including knowledge translation platforms and informal relationships between researchers and policymakers [131, 132]. Shroff et al observed that, in settings with smaller and less institutionally separated research and policy communities, personal relationships played a comparatively larger role in incorporating evidence into policy than the conventional “*two communities*” framing assumes [132]. Where science-policy interaction takes such less formalised or differently structured forms, it may be less readily captured by the search terms and conceptual frameworks that currently dominate the literature, given it does not map neatly onto the organisational forms most often described and studied. Future research that documents these forms of interaction would help to build a more complete and equitable picture of science-policy interaction during PHEs across diverse contexts.

We encourage future studies to examine science-policy dynamics in relation to non-infectious public health emergencies, such as climate and environmental crises, which are gaining urgency but remain underrepresented in the literature and in frameworks like the PHEIC.

Applying an interdisciplinary lens, we included peer-reviewed social and political science publications and thus broadened the analytical perspectives of our phenomenon of interest. However, our search relied on predefined terms that tend to be fuzzy and ill-defined in some disciplines. This might have led to a blind spot regarding publications that used other (discipline-specific) terms.

Scientific advisory systems are inevitably located in a specific political culture that determines ways, forms, and processes of advice. In this scoping review, we aimed to report on generalisable aspects of SACs in emergency settings, however, we could not account for and reflect political cultures determining specifics of scientific advice.

As with all evidence syntheses, this review is limited to documented sources identified through systematic searches. Science-policy interactions may occur in forms of engagement that are not publicly reported or published. Consequently, the findings reflect the published evidence base and may not fully capture the extent or diversity of science-policy interactions that occur in practice during PHEICs. Notably, the predominance of high-income country settings in the included literature persisted despite a search without language or reginal filters, suggesting that this pattern reflects the composition of the available evidence base rather than the language scope of our search alone.

We did not assess the quality of included articles, whether it concerned the study design or accuracy of results. In addition, articles published and disseminated during pandemics can lead to ‘premature evaluation’ with articles published without adequate peer review and scrutiny [133]. This potentially holds true as well for some articles included in this review.

The role of the media (social and traditional) in science-policy-interaction was beyond the scope of this study. In emergency settings, media play a substantial role both in the communication with the public and in communication between science and policy. These aspects could and should be addressed in future research.

Predefined procedures described in the articles may not represent the entirety of predefined procedures applied in practice, due to a publication bias. There might be further procedures (e.g., models or frameworks) in use that are not reported upon published peer-reviewed scientific literature.

## Conclusions

This scoping review explored how science-policy interactions during public health emergencies —particularly infectious disease outbreaks designated as PHEICs —are represented in the literature. Our findings indicate that formal mechanisms, particularly Scientific Advisory Committees (SACs), dominate the discourse. However, informal interactions also play a critical, albeit underexplored, role. The COVID-19 pandemic has spurred increased scholarly attention to science-policy relations, yet persistent challenges remain, including issues of transparency, independence, role ambiguity, and disciplinary imbalance.

To strengthen science-policy-interaction in future emergencies, it is essential to improve the institutional design of SACs, promote inclusive and interdisciplinary advisory structures, and acknowledge the importance of informal networks. Additionally, greater focus is needed on underrepresented regions and on non-infectious emergencies such as those related to climate change. Advancing research in these areas will support the development of more equitable, effective, and resilient science-policy interfaces during crises and beyond.

## Supplementary information

Supplementary Material 1: PRISMA-ScR) Checklist

Supplementary Material 2: Full Search Strategy per Database

Supplementary Material 3: Data extraction form

Supplementary Material 4: Coding framework

Supplementary Material 5: Characteristics of included studies

Supplementary Material 6: Additional insights from extracted data

## Data Availability

All data supporting the findings of this study are available within the paper and its Supplementary Information.

## Abbreviations

COVID-19: Corona virus disease-19
PHEIC: Public Health Emergency of International Concern
SAC: Scientific Advisory Committee
LMIC: Low- and middle-income countries
WHO: World Health Organization
