# Supplementary material for: Formal and informal science-policy interaction during public health emergencies of international concern: a scoping review
Source: BMC Glob Public Health. 2026 Sep 16;4:90. doi: 10.1186/s44263-026-00325-3 (PMC13584283; doi:10.1186/s44263-026-00325-3)
Supplement: Supplementary file 1 [file 44263_2026_325_MOESM3_ESM.pdf]

Supplementary material for “Formal and Informal Science-Policy Interaction during Public Health Emergencies of International Concern: a Global Scoping Review”
File 3: Data extraction form

Data Extraction

Record ID	______________________
What type of publication is this?	◯ Peer reviewed article<br>◯ Special issue article<br>◯ Conference abstract<br>◯ Commentary<br>◯ Report<br>◯ Other
What other type of publication is this?	______________________
Where is the first author based (geographically)?	______________________
Where is the last author based (geographically)?	______________________
Is the first author affiliated to an academic research institute?	◯ Yes<br>◯ No
What is the first author's academic affiliation (Public Health, Political Science, Sociology etc.)? Please only indicate the first affiliation	______________________
What is the first author's non-academic affiliation? Please only indicate the first affiliation	______________________
Is the last author affiliated to an academic research institute?	◯ Yes<br>◯ No
What is the last author's academic affiliation (Public Health, Political Science, Sociology etc.)? Please only indicate the first affiliation.	______________________
What is the last author's non-academic affiliation? Please only indicate the first affiliation	______________________
What is the design of the study?	◯ Quantitative<br>◯ Qualitative<br>◯ Mixed methods<br>◯ n/a

What qualitative tools were used in this study?	☐ Individual interviews<br>☐ Focus groups<br>☐ Stakeholder dialogue<br>☐ Observation<br>☐ Document analysis<br>☐ Other
What other qualitative tools were used in this study?	__________________________________
Which country/countries are described?	____
What is the World Bank country classification of the country/countries described in this study?<br><br>If not indicated, check here: https://blogs.worldbank.org/opendata/new-world-bank-country-classifications-income-level-2022-2023	☐ Low income<br>☐ Lower-middle income<br>☐ Upper-middle income<br>☐ High income
What is the date of emergency situation described? (Timespan year-year OR year)	____
Which infectious disease/virus respectively is/are described in this study?	☐ COVID-19<br>☐ H1N1/Swine flu<br>☐ H5N1/Avian flu<br>☐ MERS<br>☐ Zika<br>☐ HIV (< 2000)<br>☐ Ebola<br>☐ Other
What other infectious disease was described in this study?	____
For knowledge exchange in emergencies this study provides a ...	☐ Model<br>☐ Framework<br>☐ Toolbox<br>☐ Other
What other forms of knowledge exchange are described in this study? (copy the paragraph where authors describe it)	____
What perspectives on the science-policy-interaction are (re)presented in this paper?	☐ Science<br>☐ Decision-maker<br>☐ both<br>☐ n/a
This study describes personal interaction (as in direct in person, virtual meetings etc.) of researchers and decision-makers	◯ Yes<br>◯ No

What form of interaction is described in this study?	__________________________________
On which level of government did this interaction take place?	☐ Local<br>☐ Provincial<br>☐ Regional<br>☐ National<br>☐ International<br>☐ n/a
What was the purpose of interaction from the author's perspective?	☐ Knowledge generation<br>☐ Knowledge synthesis<br>☐ Knowledge dissemination<br>☐ Knowledge evaluation<br>☐ n/a
Do the authors describe the content of the scientific advice shared with decision-makers?	◯ Yes<br>◯ No
What is the content of the scientific advice shared with decision-makers? Please copy the parts where authors describe it	__________________________________
In what phase of the policy cycle did this interaction take place?	☐ Agenda setting<br>☐ Policy formulation<br>☐ Policy adoption<br>☐ Policy implementation<br>☐ Evaluation<br>☐ n/a
Does this study describe formal forms of science-policy-collaboration (e.g., expert committee, policy advice organisation)?	◯ Yes<br>◯ No
What kind of forms of science-policy collaboration are described in this study?	☐ Expert committee<br>☐ Scientific advisory group<br>☐ Ad-hoc established committee<br>☐ Policy advice organisation<br>☐ Other
Does this study present information on how the science-policy collaboration participants were selected/installed? (copy the parts where authors describe)	__________________________________
What other forms of collaboration is described in this study?	__________________________________
Do the authors indicate their perception of the interaction (for instance, was it fruitful, challenging etc.)?	◯ Yes<br>◯ No
How do the authors desrcibe their perception of the interaction? (Copy the parts where they describe it)	__________________________________

Does this study describe barriers in science-policy interaction?	◯ Yes<br>◯ No
What are the barriers mentioned in this study? (copy the part where authors describe them)	__________________________________
Does this study describe facilitators in science-policy interaction?	◯ Yes<br>◯ No
What are the facilitators mentioned in this study? (copy the part where authors describe them)	____________________________
Does this study describe and apply a specific model of science-policy interaction ?	◯ Yes<br>◯ No
Which model is described and applied in this study? (copy the part where authors describe them):	__________________________________
What formats were used for knowledge exchange?	☐ Formal Report<br>☐ Digital Platform<br>☐ Personal Exchange (telephone, round tables etc.)<br>☐ n/a<br>☐ Other
What other formats were used for knowledge exchange?	__________________________________
Does this paper give information on knowledge push (science driven) or knowledge pull (government driven)?	☐ knowledge push (science driven)<br>☐ knowledge pull (government driven)<br>☐ both<br>☐ n/a
