# Supplementary material for: Formal and informal science-policy interaction during public health emergencies of international concern: a scoping review
Source: BMC Glob Public Health. 2026 Sep 16;4:90. doi: 10.1186/s44263-026-00325-3 (PMC13584283; doi:10.1186/s44263-026-00325-3)
Supplement: Supplementary file 3 [file 44263_2026_325_MOESM2_ESM.docx]

**Supplementary material for “Formal and Informal Science-Policy Interaction during Public Health Emergencies of International Concern: a Global Scoping Review”**

**Additional File 2. Full Search Strategy per Database**

Research question:
"What are approaches, tools and guidelines for knowledge translation and interaction between researchers and decision makers in public health emergency response?" A Scoping review.

Overview of database sources & results
Original search: 22.04.2022; Update 1: 22.09.2023; Update 2: 10.03.2026.
*Records shown are cumulative totals across all three search dates.*

| **Database searched** | **Platform** | **Database coverage** | **Records** | **Records after duplicates removed** |
| --- | --- | --- | --- | --- |
| **Embase.com** | Elsevier | 1974 - Present | 3’253 |  |
| **MEDLINE ALL** | Ovid | 1946 - Present | 3’580 |  |
| **Cochrane Library** | Wiley | 1992 - Present | 111 |  |
| **Social Sciences Citation Index Expanded (SSCI)** | Clarivate | 1900 - Present | 2’704 |  |
| **ERIC** | OvidSP | 1973 - Present | 298 |  |
| **Global Health** | OvidSP | 1900 - Present | 1’862 |  |
| **Google Scholar** |  | Search engine | 200  top ranked hits |  |
| **Total** | | | 12’008 | **8’057** |

3’951 duplicate records were removed using Deduklick, a validated automated deduplication algorithm^[1]
[1]^Borissov, N.; Haas, Q.; Minder, B.; Kopp-Heim, D.; von Gernler, M.; Janka, H.; Teodoro, D.; Amini, P. Reducing systematic review burden using Deduklick: A novel, automated, reliable, and explainable deduplication algorithm to foster medical research. Syst. Rev. 2022, 11, 172.
Note:
Deduplication was performed on the pooled dataset rather than per database; therefore, no per-database figures are reported.

**Search concepts and combinations**

**Embase.com (via Elsevier)** Combination of search steps: 1) AND 2) AND 3) AND 4) – NOT 5)

**1) Decision Making, Health Care Policy, Crisis Management**

('decision making'/de OR 'health care policy'/de OR 'disaster preparedness'/de OR 'preparedness'/de OR 'crisis management'/de OR 'disaster management'/exp OR 'risk management'/de OR 'response'/de OR 'disaster response'/de OR 'disaster mitigation'/de OR 'situational awareness'/de OR 'decision support system'/de OR ('decision mak*' OR decisionmak* OR 'decision support' OR ((policy OR policies) NEAR/3 (advice* OR advis* OR develop* OR decision* OR maker* OR making* OR communicat*)) OR policymak* OR ('public health' NEAR/2 (polic* OR politic* OR decision*)) OR 'policy entrepreneur*' OR 'policy communication' OR (science NEXT/2 policy NEXT/2 (communica* OR interfac*)) OR 'health care polic*' OR 'health polic*' OR 'politics of health' OR 'health in all polic*' OR 'political determinants of health' OR 'health system administrat*' OR 'health services administrat*' OR preparedness OR ((emergenc* OR disaster* OR pandemi* OR crisis) NEAR/3 (response* OR management* OR leadership* OR governance)) OR 'pandemic plan*' OR 'organization and management' OR 'multilevel governance' OR 'disaster risk reduction' OR 'situational awareness'):ab,ti,kw)

**2) Knowledge translation, Evidence (scientific, research)**

('evidence based practice'/de OR 'knowledge transfer'/de OR 'knowledge translation'/de OR 'science'/de OR 'translational science'/de OR 'implementation science'/de OR ('scientific evidence' OR 'research evidence' OR 'science based' OR ('evidence base*' NEAR/3 (policy OR policies)) OR 'evidence for policy' OR 'evidence-based practice*' OR 'evidence-inform*' OR 'evidence uptak*' OR 'evidence use' OR 'evidence usage' OR ((knowledge OR research) NEXT/1 (utiliz* OR utilis* OR uptak*)) OR 'scientific knowledge' OR evidentiary OR ((scientific OR expert*) NEAR/3 (advice* OR advis* OR recommendation*)) OR 'implementation science' OR (implement* NEAR/3 (science OR scientific OR research)) OR 'improvement science' OR (science-policy NEAR/2 interface) OR 'science and policy' OR 'epistemic learning' OR 'policy learning' OR (knowledge NEAR/2 (transfer* OR translat* OR exchang* OR disseminat* OR broke* OR use*)) OR ((evidence* OR research*) NEAR/2 (transfer* OR translat* OR exchang* OR disseminat* OR use*)) OR 'evidence to action' OR 'evidence to practice' OR 'research to action' OR 'research into action' OR 'knowledge into action*' OR 'information transparency' OR expertise OR 'expert* meeting*' OR 'expert* committee*' OR 'external experts' OR 'scientific expert*' OR 'scientific communit*' OR 'health scientist*' OR cross-cutting OR transdisciplinar* OR 'scientific uncertaint*'):ab,ti,kw)

**3) Public Health emergencies**

('pandemic influenza'/de OR 'pandemic'/de OR 'health crisis'/de OR 'epidemic'/de OR 'outbreak'/de OR 'emerging infectious disease'/de OR 'Q fever'/de OR 'influenza A (H5N1)'/exp OR 'influenza A (H1N1)'/exp OR 'Middle East respiratory syndrome coronavirus'/exp OR 'Zika fever'/de OR 'poliomyelitis'/de OR 'coronavirus disease 2019'/exp OR 'Severe acute respiratory syndrome coronavirus 2'/exp OR 'Coronavirus infection'/de OR 'SARS-related coronavirus'/de OR 'severe acute respiratory syndrome'/de OR 'Ebola hemorrhagic fever'/de OR (pandemi* OR 'health emergenc*' OR 'health cris*' OR 'humanitarian cris*' OR 'public health emergencies of international concern' OR 'PHEIC' OR (emerging NEXT/2 disease*) OR 'health threat*' OR 'health disaster*' OR epidemic* OR outbreak* OR 'crisis zone*' OR 'global health security' OR Q-fever OR H5N1 OR H1N1 OR 'swine flu' OR '1918 flu' OR 'Spanish influenza' OR 'Spanish flu' OR 'Middle East Respiratory Syndrome' OR MERS OR polio* OR Zika OR Ebola OR COVID-19 OR COVID19 OR 'corona crisis' OR 'corona virus*' OR coronavirus* OR 'severe acute respiratory syndrome' OR SARS):ab,ti,kw)

**4) Approaches, tools, guidelines, frameworks**

('conceptual framework'/de OR 'program development'/de OR (approach OR approaches OR tool OR tools OR platform* OR guideline* OR recommendat* OR consensus OR framework* OR framing OR instrument* OR operational* OR rules OR interventions OR model OR models OR standard* OR adopt* OR implement* OR integration* OR institutionalization* OR institutionalisation* OR ((program* OR process*) NEAR/3 (develop* OR plan OR plans OR plann*))):ab,ti,kw)

**5) Filters: exclude animal studies**

NOT ([animals]/lim NOT [humans]/lim)

**Detailed search strategies per database**

**Embase.com** (via Elsevier)

('decision making'/de OR 'health care policy'/de OR 'disaster preparedness'/de OR 'preparedness'/de OR 'crisis management'/de OR 'disaster management'/exp OR 'risk management'/de OR 'response'/de OR 'disaster response'/de OR 'disaster mitigation'/de OR 'situational awareness'/de OR 'decision support system'/de OR ('decision mak*' OR decisionmak* OR 'decision support' OR ((policy OR policies) NEAR/3 (advice* OR advis* OR develop* OR decision* OR maker* OR making* OR communicat*)) OR policymak* OR ('public health' NEAR/2 (polic* OR politic* OR decision*)) OR 'policy entrepreneur*' OR 'policy communication' OR (science NEXT/2 policy NEXT/2 (communica* OR interfac*)) OR 'health care polic*' OR 'health polic*' OR 'politics of health' OR 'health in all polic*' OR 'political determinants of health' OR 'health system administrat*' OR 'health services administrat*' OR preparedness OR ((emergenc* OR disaster* OR pandemi* OR crisis) NEAR/3 (response* OR management* OR leadership* OR governance)) OR 'pandemic plan*' OR 'organization and management' OR 'multilevel governance' OR 'disaster risk reduction' OR 'situational awareness'):ab,ti,kw) AND ('evidence based practice'/de OR 'knowledge transfer'/de OR 'knowledge translation'/de OR 'science'/de OR 'translational science'/de OR 'implementation science'/de OR ('scientific evidence' OR 'research evidence' OR 'science based' OR ('evidence base*' NEAR/3 (policy OR policies)) OR 'evidence for policy' OR 'evidence-based practice*' OR 'evidence-inform*' OR 'evidence uptak*' OR 'evidence use' OR 'evidence usage' OR ((knowledge OR research) NEXT/1 (utiliz* OR utilis* OR uptak*)) OR 'scientific knowledge' OR evidentiary OR ((scientific OR expert*) NEAR/3 (advice* OR advis* OR recommendation*)) OR 'implementation science' OR (implement* NEAR/3 (science OR scientific OR research)) OR 'improvement science' OR (science-policy NEAR/2 interface) OR 'science and policy' OR 'epistemic learning' OR 'policy learning' OR (knowledge NEAR/2 (transfer* OR translat* OR exchang* OR disseminat* OR broke* OR use*)) OR ((evidence* OR research*) NEAR/2 (transfer* OR translat* OR exchang* OR disseminat* OR use*)) OR 'evidence to action' OR 'evidence to practice' OR 'research to action' OR 'research into action' OR 'knowledge into action*' OR 'information transparency' OR expertise OR 'expert* meeting*' OR 'expert* committee*' OR 'external expert*' OR 'scientific expert*' OR 'scientific communit*' OR 'health scientist*' OR cross-cutting OR transdisciplinar* OR 'scientific uncertaint*'):ab,ti,kw) AND ('pandemic influenza'/de OR 'pandemic'/de OR 'health crisis'/de OR 'epidemic'/de OR 'outbreak'/de OR 'emerging infectious disease'/de OR 'Q fever'/de OR 'influenza A (H5N1)'/exp OR 'influenza A (H1N1)'/exp OR 'Middle East respiratory syndrome coronavirus'/exp OR 'Zika fever'/de OR 'poliomyelitis'/de OR 'coronavirus disease 2019'/exp OR 'Severe acute respiratory syndrome coronavirus 2'/exp OR 'Coronavirus infection'/de OR 'SARS-related coronavirus'/de OR 'severe acute respiratory syndrome'/de OR 'Ebola hemorrhagic fever'/de OR (pandemi* OR 'health emergenc*' OR 'health cris*' OR 'humanitarian cris*' OR 'public health emergencies of international concern' OR 'PHEIC' OR (emerging NEXT/2 disease*) OR 'health threat*' OR 'health disaster*' OR epidemic* OR outbreak* OR 'crisis zone*' OR 'global health security' OR Q-fever OR H5N1 OR H1N1 OR 'swine flu' OR '1918 flu' OR 'Spanish influenza' OR 'Spanish flu' OR 'Middle East Respiratory Syndrome' OR MERS OR polio* OR Zika OR Ebola OR COVID-19 OR COVID19 OR 'corona crisis' OR 'corona virus*' OR coronavirus* OR 'severe acute respiratory syndrome' OR SARS):ab,ti,kw) AND ('conceptual framework'/de OR 'program development'/de OR (approach OR approaches OR tool OR tools OR platform* OR guideline* OR recommendat* OR consensus OR framework* OR framing OR instrument* OR operational* OR rules OR interventions OR model OR models OR standard* OR adopt* OR implement* OR integration* OR institutionalization* OR institutionalisation* OR ((program* OR process*) NEAR/3 (develop* OR plan OR plans OR plann*))):ab,ti,kw) NOT ([animals]/lim NOT [humans]/lim)

**Medline ALL (Ovid)**
Ovid MEDLINE(R) ALL

(decision making/ OR health policy/ OR exp Policy Making/ OR disaster planning/ OR risk management/ OR awareness/ OR decision support techniques/ OR (decision mak* OR decisionmak* OR decision support OR ((policy OR policies) ADJ3 (advice* OR advis* OR develop* OR decision* OR maker* OR making* OR communicat*)) OR policymak* OR (public health ADJ2 (polic* OR politic* OR decision*)) OR policy entrepreneur* OR policy communication OR (science ADJ2 policy ADJ2 (communica* OR interfac*)) OR health care polic* OR health polic* OR politics of health OR health in all polic* OR political determinants of health OR health system administrat* OR health services administrat* OR preparedness OR ((emergenc* OR disaster* OR pandemi* OR crisis) ADJ3 (response* OR management* OR leadership* OR governance)) OR pandemic plan* OR "organization and management" OR "multilevel governance" OR disaster risk reduction OR situational awareness).ab,ti,kf.) AND (evidence-based practice/ OR science/ OR implementation science/ OR knowledge translation/ OR (scientific evidence OR research evidence OR science based OR (evidence base* ADJ3 (policy OR policies)) OR "evidence for policy" OR evidence-based practice* OR evidence-inform* OR evidence uptak* OR "evidence use" OR "evidence usage" OR ((knowledge OR research) ADJ (utiliz* OR utilis* OR uptak*)) OR scientific knowledge OR evidentiary OR ((scientific OR expert*) ADJ3 (advice* OR advis* OR recommendation*)) OR "implementation science" OR (implement* ADJ3 (science OR scientific OR research)) OR "improvement science" OR (science-policy ADJ2 interface) OR "science and policy" OR "epistemic learning" OR "policy learning" OR (knowledge ADJ2 (transfer* OR translat* OR exchang* OR disseminat* OR broke* OR use*)) OR ((evidence* OR research*) ADJ2 (transfer* OR translat* OR exchang* OR disseminat* OR use*)) OR "evidence to action" OR "evidence to practice" OR "research to action" OR "research into action" OR "knowledge into action*" OR information transparency OR expertise OR expert* meeting* OR expert* committee* OR "external expert*" OR "scientific expert*" OR scientific communit* OR health scientist* OR cross-cutting OR transdisciplinar* OR "scientific uncertaint*").ab,ti,kf.) AND (pandemics/ OR epidemics/ OR disease outbreaks/ OR Communicable Diseases, Emerging/ OR Q fever/ OR Influenza A Virus, H1N1 Subtype/ OR Influenza A Virus, H5N1 Subtype/ OR Middle East respiratory syndrome coronavirus/ OR Zika virus infection/ OR poliomyelitis/ OR COVID-19/ OR SARS-CoV-2/ OR Coronavirus infections/ OR SARS Virus/ OR severe acute respiratory syndrome/ OR Hemorrhagic Fever, Ebola/ OR (pandemi* OR health emergenc* OR health cris* OR humanitarian cris* OR "public health emergencies of international concern" OR PHEIC OR (emerging ADJ2 disease*) OR health threat* OR health disaster* OR epidemic* OR outbreak* OR crisis zone* OR "global health security" OR Q-fever OR H5N1 OR H1N1 OR swine flu OR 1918 flu OR Spanish influenza OR Spanish flu OR Middle East Respiratory Syndrome OR MERS OR polio* OR Zika OR Ebola OR COVID-19 OR COVID19 OR "corona crisis" OR corona virus* OR coronavirus* OR severe acute respiratory syndrome OR SARS).ab,ti,kf.) AND (program development/ OR (approach OR approaches OR tool OR tools OR platform* OR guideline* OR recommendat* OR consensus OR framework* OR framing OR instrument* OR operational* OR rules OR interventions OR model OR models OR standard* OR adopt* OR implement* OR integration* OR institutionalisation* OR institutionalization* OR ((program* OR process*) ADJ3 (develop* OR plan OR plans OR plann*))).ab,ti,kf.) NOT (exp animals/ NOT humans/)

**Cochrane Library** (via Wiley)

((decision NEXT mak* OR decisionmak* OR "decision support" OR ((policy OR policies) NEAR/3 (advice* OR advis* OR develop* OR decision* OR maker* OR making* OR communicat*)) OR policymak* OR ("public health" NEAR/2 (polic* OR politic* OR decision*)) OR policy NEXT entrepreneur* OR "policy communication" OR (science NEXT/2 policy NEXT/2 (communica* OR interfac*)) OR health NEXT care NEXT polic* OR health NEXT polic* OR "politics of health" OR "health in all policy" OR "health in all policies" OR "political determinants of health" OR ("health system" NEXT administrat*) OR ("health services" NEXT administrat*) OR preparedness OR ((emergenc* OR disaster* OR pandemi* OR crisis) NEAR/3 (response* OR management* OR leadership* OR governance)) OR pandemic NEXT plan* OR "organization and management" OR "disaster risk reduction" OR "multilevel governance" OR "situational awareness"):ab,ti,kw) AND ((evidence NEXT base* NEXT/3 polic* OR "evidence for policy" OR evidence NEXT based NEXT practice* OR evidence NEXT inform* OR evidence NEXT uptak* OR "evidence use" OR "evidence usage" OR ((knowledge OR research) NEXT/1 (utiliz* OR utilis* OR uptak*)) OR "scientific knowledge" OR evidentiary OR ((scientific OR expert*) NEAR/3 (advice* OR advis* OR recommendation*)) OR "implementation science" OR (implement* NEAR/3 (science OR scientific OR research)) OR "improvement science" OR (science-policy NEAR/2 interface) OR "science and policy" OR "epistemic learning" OR "policy learning" OR (knowledge NEAR/2 (transfer* OR translat* OR exchang* OR disseminat* OR broke* OR use*)) OR ((evidence* OR research*) NEAR/2 (transfer* OR translat* OR exchang* OR disseminat* OR use*)) OR "evidence to action" OR "evidence to practice" OR "research to action" OR "research into action" OR "knowledge into action" OR "information transparency" OR expertise OR expert* NEXT meeting* OR expert* NEXT committee* OR external NEXT expert* OR scientific NEXT expert* OR scientific NEXT communit* OR health NEXT scientist* OR "cross-cutting" OR transdisciplinar* OR scientific NEXT uncertaint*):ab,ti,kw) AND ((pandemi* OR health NEXT emergenc* OR health NEXT cris* OR humanitarian NEXT cris* OR "public health emergencies of international concern" OR "PHEIC" OR (emerging NEXT/2 disease*) OR health NEXT threat* OR health NEXT disaster* OR epidemic* OR outbreak* OR crisis NEXT zone* OR "global health security" OR "Q-fever" OR H5N1 OR H1N1 OR "swine flu" OR "1918 flu" OR "Spanish influenza" OR "Spanish flu" OR "Middle East Respiratory Syndrome" OR MERS OR polio* OR Zika OR Ebola OR "COVID-19" OR COVID19 OR "corona crisis" OR corona NEXT virus* OR coronavirus* OR "severe acute respiratory syndrome" OR SARS):ab,ti,kw)

**Social Sciences Citation Index Expanded (SCI-EXPANDED) – 1900-present**
(searched via: Web of Science Core Collection, Clarivate)

TS=((("decision mak*" OR decisionmak* OR "decision support" OR ((policy OR policies) NEAR/2 (advice* OR advis* OR develop* OR decision* OR maker* OR making* OR communicat*)) OR policymak* OR ("public health" NEAR/1 (polic* OR politic* OR decision*)) OR "policy entrepreneur*" OR "policy communication" OR (science NEAR/1 policy NEAR/2 (communica* OR interfac*)) OR "health care polic*" OR "health polic*" OR "politics of health" OR "health in all policy" OR "health in all policies" OR "political determinants of health" OR "health system administrat* " OR "health services administrat*" OR preparedness OR ((emergenc* OR disaster* OR pandemi* OR crisis) NEAR/2 (response* OR management* OR leadership* OR governance)) OR "pandemic plan*" OR "organization and management" OR "multilevel governance" OR "disaster risk reduction" OR "situational awareness")) AND ((("evidence base*" NEAR/3 polic*) OR "evidence for policy" OR "evidence based practice*" OR "evidence inform*" OR "evidence uptak*" OR "evidence use" OR "evidence usage" OR ((knowledge OR research) NEAR/1 (utiliz* OR utilis* OR uptak*)) OR "scientific knowledge" OR evidentiary OR ((scientific OR expert*) NEAR/2 (advice* OR advis* OR recommendation*)) OR "implementation science" OR (implement* NEAR/2 (science OR scientific OR research)) OR "improvement science" OR (science-policy NEAR/1 interface) OR "science and policy" OR "epistemic learning" OR "policy learning" OR (knowledge NEAR/1 (transfer* OR translat* OR exchang* OR disseminat* OR broke* OR use*)) OR ((evidence* OR research*) NEAR/2 (transfer* OR translat* OR exchang* OR disseminat* OR use*)) OR "evidence to action" OR "evidence to practice" OR "research to action" OR "research into action" OR "knowledge into action" OR "information transparency" OR expertise OR "expert* meeting*" OR "expert* committee*" OR "external expert*" OR "scientific expert*" OR "scientific communit*" OR "health scientist*" OR "cross-cutting" OR transdisciplinar* OR "scientific uncertaint*")) AND ((pandemi* OR "health emergenc*" OR "health cris*" OR "humanitarian cris*" OR "public health emergencies of international concern" OR "PHEIC" OR (emerging NEAR/2 disease*) OR "health threat*" OR "health disaster*" OR epidemic* OR outbreak* OR "crisis zone*" OR "global health security" OR "Q-fever" OR H5N1 OR H1N1 OR "swine flu" OR "1918 flu" OR "Spanish influenza" OR "Spanish flu" OR "Middle East Respiratory Syndrome" OR MERS OR polio* OR Zika OR Ebola OR "COVID-19" OR COVID19 OR "corona crisis" OR "corona virus*" OR coronavirus* OR "severe acute respiratory syndrome" OR SARS)) AND ((approach OR approaches OR tool OR tools OR platform* OR guideline* OR recommendat* OR consensus OR framework* OR framing OR instrument* OR operational* OR rules OR interventions OR model OR models OR standard* OR adopt* OR implement* OR integration* OR institutionalization* OR institutionalisation* OR ((program* OR process*) NEAR/2 (develop* OR plan OR plans OR plann*)))))

**ERIC (OvidSP)**

(decision making/ OR public policy/ OR policy formation/ OR emergency programs/ OR crisis management/ OR risk management/ OR (decision mak* OR decisionmak* OR decision support OR ((policy OR policies) ADJ3 (advice* OR advis* OR develop* OR decision* OR maker* OR making* OR communicat*)) OR policymak* OR (public health ADJ2 (polic* OR politic* OR decision*)) OR policy entrepreneur* OR policy communication OR (science ADJ2 policy ADJ2 (communica* OR interfac*)) OR health care polic* OR health polic* OR politics of health OR health in all polic* OR political determinants of health OR health system administrat* OR health services administrat* OR preparedness OR ((emergenc* OR disaster* OR pandemi* OR crisis) ADJ3 (response* OR management* OR leadership* OR governance)) OR pandemic plan* OR "organization and management" OR "multilevel governance" OR disaster risk reduction OR situational awareness).tw.) AND (evidence based practice/ OR evidence/ OR sciences/ OR expertise/ OR scientific research/ OR program implementation/ OR research utilization/ OR information dissemination/ OR (scientific evidence OR research evidence OR science based OR (evidence base* ADJ3 (policy OR policies)) OR "evidence for policy" OR evidence-based practice* OR evidence-inform* OR evidence uptak* OR "evidence use" OR "evidence usage" OR ((knowledge OR research) ADJ (utiliz* OR utilis* OR uptak*)) OR scientific knowledge OR evidentiary OR ((scientific OR expert*) ADJ3 (advice* OR advis* OR recommendation*)) OR "implementation science" OR (implement* ADJ3 (science OR scientific OR research)) OR "improvement science" OR (science-policy ADJ2 interface) OR "science and policy" OR ((epistemic or policy) ADJ3 learning) OR (knowledge ADJ2 (transfer* OR translat* OR exchang* OR disseminat* OR broke* OR use*)) OR ((evidence* OR research*) ADJ2 (transfer* OR translat* OR exchang* OR disseminat* OR use*)) OR "evidence to action" OR "evidence to practice" OR "research to action" OR "research into action" OR "knowledge into action*" OR information transparency OR expertise OR expert* meeting* OR expert* committee* OR "external expert*" OR scientific expert* OR scientific communit* OR health scientist* OR cross-cutting OR transdisciplinar* OR "scientific uncertaint*").tw.) AND (pandemics/ OR disease control/ OR communicable diseases/ OR COVID-19/ OR (pandemi* OR health emergenc* OR health cris* OR humanitarian cris* OR "public health emergencies of international concern" OR PHEIC OR (emerging ADJ2 disease*) OR health threat* OR health disaster* OR epidemic* OR outbreak* OR crisis zone* OR "global health security" OR Q-fever OR H5N1 OR H1N1 OR swine flu OR 1918 flu OR Spanish influenza OR Spanish flu OR Middle East Respiratory Syndrome OR MERS OR polio* OR Zika OR Ebola OR COVID-19 OR COVID19 OR "corona crisis" OR corona virus* OR coronavirus* OR severe acute respiratory syndrome OR SARS).tw.) AND (program development/ OR (approach OR approaches OR tool OR tools OR platform* OR guideline* OR recommendat* OR consensus OR framework* OR framing OR instrument* OR operational* OR rules OR interventions OR model OR models OR standard* OR adopt* OR implement* OR integration* OR institutionalisation* OR institutionalization* OR ((program* OR process*) ADJ3 (develop* OR plan OR plans OR plann*))).tw.)

**Global Health** (OvidSP) **–** *Subscription cancelled by the end of Nov 2025*

(decision making/ OR health policy/ OR awareness/ OR (decision mak* OR decisionmak* OR decision support OR ((policy OR policies) ADJ3 (advice* OR advis* OR develop* OR decision* OR maker* OR making* OR communicat*)) OR policymak* OR (public health ADJ2 (polic* OR politic* OR decision*)) OR policy entrepreneur* OR policy communication OR (science ADJ2 policy ADJ2 (communica* OR interfac*)) OR health care polic* OR health polic* OR politics of health OR health in all polic* OR political determinants of health OR health system administrat* OR health services administrat* OR preparedness OR ((emergenc* OR disaster* OR pandemi* OR crisis) ADJ3 (response* OR management* OR leadership* OR governance)) OR pandemic plan* OR "organization and management" OR "multilevel governance" OR disaster risk reduction OR situational awareness).tw.) AND (knowledge/ OR "diffusion of information"/ OR (scientific evidence OR research evidence OR science based OR (evidence base* ADJ3 (policy OR policies)) OR "evidence for policy" OR evidence-based practice* OR evidence-inform* OR evidence uptak* OR "evidence use" OR "evidence usage" OR ((knowledge OR research) ADJ (utiliz* OR utilis* OR uptak*)) OR scientific knowledge OR evidentiary OR ((scientific OR expert*) ADJ3 (advice* OR advis* OR recommendation*)) OR "implementation science" OR (implement* ADJ3 (science OR scientific OR research)) OR "improvement science" OR (science-policy ADJ2 interface) OR "science and policy" OR "epistemic learning" OR "policy learning" OR (knowledge ADJ2 (transfer* OR translat* OR exchang* OR disseminat* OR broke* OR use*)) OR ((evidence* OR research*) ADJ2 (transfer* OR translat* OR exchang* OR disseminat* OR use*)) OR "evidence to action" OR "evidence to practice" OR "research to action" OR "research into action" OR "knowledge into action*" OR information transparency OR expertise OR expert* meeting* OR expert* committee* OR "external expert*" OR scientific expert* OR scientific communit* OR health scientist* OR cross-cutting OR transdisciplinar* OR "scientific uncertaint*").tw.) AND (pandemics/ OR epidemics/ OR outbreaks/ OR emerging infectious diseases/ OR Q fever/ OR Influenza A Virus/ OR Middle East respiratory syndrome coronavirus/ OR Zika virus/ OR Zika fever/ OR poliomyelitis/ OR "coronavirus disease 2019"/ OR exp severe acute respiratory syndrome coronavirus/ OR ebola haemorrhagic fever/ OR (pandemi* OR health emergenc* OR health cris* OR humanitarian cris* OR "public health emergencies of international concern" OR PHEIC OR (emerging ADJ2 disease*) OR health threat* OR health disaster* OR epidemic* OR outbreak* OR crisis zone* OR "global health security" OR Q-fever OR H5N1 OR H1N1 OR swine flu OR 1918 flu OR Spanish influenza OR Spanish flu OR Middle East Respiratory Syndrome OR MERS OR polio* OR Zika OR Ebola OR COVID-19 OR COVID19 OR "corona crisis" OR corona virus* OR coronavirus* OR severe acute respiratory syndrome OR SARS).tw.) AND (program development/ OR (approach OR approaches OR tool OR tools OR platform* OR guideline* OR recommendat* OR consensus OR framework* OR framing OR instrument* OR operational* OR rules OR interventions OR model OR models OR standard* OR adopt* OR implement* OR integration* OR institutionalisation* OR institutionalization* OR ((program* OR process*) ADJ3 (develop* OR plan OR plans OR plann*))).tw.)

**Google Scholar Original search** (first 200 results, sorted by relevance)

| **#** | **Query for Original search** |
| --- | --- |
| 1 | "decision\|policy making\|makers"\|policymaking\|governance\|preparedness\|"emergency\|crisis\|disaster management" "evidence based\|informed\|uptake\|use\|usage\|policy"\|"scientific\|expert advice"\|"knowledge transfer\|translation\|exchange" outbreak\|pandemic |

Note: Search field allows only 252 characters, therefore search string is shortened.

**Google Scholar Update searches** (first 200 results, sorted by relevance)
For the updated searches, we designed a simpler search string. We retrieved the first 200 results, sorted by relevance, and restricted them to publications from 2023 to 2026. Due to Google Scholar's character limitations and the lack of transparency regarding its ranking algorithm, this approach is a pragmatic compromise that aligns with established guidelines for using Google Scholar in evidence reviews (Haddaway et al., 2015; Gusenbauer & Haddaway, 2020).

| **#** | **Query for Update search** |
| --- | --- |
| 1 | science\|scientific\|evidence\|knowledge\|expert policy\|policymaking\|political\|policies\|advice\|advisory\|decision interaction\|interactions\|translation\|response “public health”\|”global health” emergency\|emergencies\|pandemic\|outbreaks\|crisis |

*Haddaway, N.R., Collins, A.M., Coughlin, D. and Kirk, S. (2015) The Role of Google Scholar in Evidence Reviews and Its Applicability to Grey Literature Searching. PLOS ONE, 10(9), e0138237. doi:10.1371/journal.pone.0138237*

*Gusenbauer, M. and Haddaway, N.R. (2020) Which academic search systems are suitable for systematic reviews or meta-analyses? Evaluating retrieval qualities of Google Scholar, PubMed, and 26 other resources. Research Synthesis Methods, 11(2), 181–217. DOI: https://doi.org/10.1002/jrsm.1378*

For the bulk import of references from Google Scholar into EndNote, we used the software Publish or Perish (Harzing; RRID: SCR_005968).
