# Supplementary material for: Formal and informal science-policy interaction during public health emergencies of international concern: a scoping review
Source: BMC Glob Public Health. 2026 Sep 16;4:90. doi: 10.1186/s44263-026-00325-3 (PMC13584283; doi:10.1186/s44263-026-00325-3)
Supplement: Supplementary file 4 [file 44263_2026_325_MOESM6_ESM.docx]

Supplementary material for “Formal and Informal Science-Policy Interaction during Public Health Emergencies of International Concern: a global scoping Review”

File 6: Additional insights from extracted data

I. Characteristics of PHEIC influencing science-policy interaction

The articles referred to established definitions of emergency settings as situations that are demanding in various aspects: high pressure and time constraining, governmental administrations perceived as slow, understaffed and unprepared—to name only a few such characteristics (1-16). Exhaustion complicated any science-policy interaction as engagement waned several months into the COVID-19 pandemic when preventive measures relaxed and governmental interest diminished (17). Finally, already prevailing inequities in access to resources intensified and added additional pressure on researchers in search of evidence applicable in low- and middle-income countries contexts (18). In line with this, existing challenges like short-term political attention and unstable funding undermined the independence and effectiveness of the scientific policy advice during the COVID-19 pandemic, as described for the Australian case (19).

II. Composition and Omissions in SACs

The composition of SACs typically reflected a range of expertise within the biomedical and natural sciences, including disciplines such as virology, epidemiology, statistics, and clinical medicine. In many cases, committees were composed either entirely or predominantly of experts from these scientific domains (4, 9, 11, 14, 20-36). Rozenblum reported on France, where an SAC was ‘mostly made up of medical doctors, thus giving greater weight to clinical judgements than public health reasoning in the decision-making process’ (11).

Although many statisticians were anxious to contribute to efforts to cope with COVID-19, most of them found they had almost no ability to influence policy. At the same time, leading researchers in disciplines not directly related to statistics and epidemiology were approached to contribute and influence policy decisions (37).

Over the duration of the emergency settings, the composition of SACs in some instances was in flux: people leaving and joining SACs due to conflicts or emerging topics calling for additional expertise appeared to be a common denominator (4, 7, 13, 25, 27, 34, 38, 39).

In some cases, the SACs included experts from diverse disciplines such as medical science, data science, sociology, and anthropology (11, 15, 21, 39-41). Nevertheless, the social sciences (6), psychology (31), and economics and behavioural science (15) usually did not have a seat at the table.

Moreover, none of the included articles mentioned experts from the humanities, nor did any report inter- or transdisciplinary work in SACs. Articles in UK (1, 42, 43), Italy (44), Australia (45), and India (46) identified clear knowledge hierarchies and power relations that lay bare a general perception of ‘political and sociological evaluations of social scientists and ethicists,’ that ‘overlap […] with policymakers’ assessment and core competencies,’ which therefore are ‘redundant’ (44). Eichenberger et al. showed that in Switzerland the recommendations by the expert groups on “Economics” and “Ethics, Legal, and Social” were less likely to be implemented than those made by other expert groups (36). Finally, for the Italian case, two paper reported majority of men in SACs revealing a considerable gender imbalance in SACs (44, 47). The unpaid workload of SAC commitment was intense. What’s more, enrolled female experts were disproportionately burdened by caregiving responsibilities and limited travel flexibility, which were further intensified during the COVID-19 pandemic, while male experts tended to be wealthy and without further burdens beyond the job. For female researchers, SAC participation made comparatively crushing demands (44). Wabnitz et al. raised the question of whether members of a politically relevant guideline development panel should be democratically elected (48).

III. Interaction Enablers

A ‘clearly defined chain of command and organizational structure, effective resource management, and advanced planning’ were reported as ‘important aspects of an emergency response’ (33). Several articles discussed the necessity of two-way communication between policymakers and modellers to deliberate key priorities and questions as there was often a trade-off between what policymakers wanted and what was possible with the available data (8, 10, 11, 13, 49, 50). Yet, this can also be a time consuming and tiring process (51).

SACs improved communication by framing their advice in already widely accepted concepts (25, 52), adopting user friendly formats and precise queries to be used by policymakers (2, 6, 10, 16, 49, 51, 53-56), and generally ‘translating the evidence’ (16, 25, 41).

Embeddedness of both researchers and policymakers within each other’s structures facilitated collaboration, mutual understanding of processes (10). Vickery et al. thus present ‘education of scientists and policy-makers about each other’s needs, perspectives and constraints to [Evidence Informed Decision Making] EIDM’ as a ‘fruitful approach to promoting priorisation of scientific evidence’ (13). Furthermore, co-creational approaches fostered the acceptability and utilization of evidence in decision-making processes. Finally, presenting a unanimous message (34) ‘so that their advice is more coherent and less susceptible to attack or denigration’ (25) smoothed communication and evidence uptake. Rankin et al. described a successful priority-setting roundtable of senior leaders from clinical health services, policy makers, and the research community (57). A give-and-take mindset was presented as fostering collaboration despite constant budgetary pressures (25) as well as keeping an eye on inclusion of broad expertise in SACs (13).

In some documented science-policy interactions, time played a crucial role in deepening and sustaining good working relationships; collaboration improved with the passage of time (8, 50, 51, 58). Trust was fostered through a variety of factors: the curiosity and motivation of researchers and policymakers to tackle complexity (17), availability (29, 59), regular meetings (3, 15), rapid responses (55), credibility of research institutions through constant communication with policy-makers (10), and financial resources to fund intense support (5, 60)(30*, 33*).

1.	Atkinson P, Mableson H, Sheard S, Martindale AM, Solomon T, Borek A, et al. How did UK policymaking in the COVID-19 response use science? Evidence from scientific advisers. Evidence & Policy. 2022;18(4):633–50.

2.	Bahr T, Klein N, Fehr A, Fischer HT, Hanefeld J, Iskandarani D, et al. Providing strategic information during a public health emergency: lessons learned during the COVID-19 pandemic in Germany. Evidence & Policy. 2023;19(4):606–12.

3.	Bruat C, Monnet E, Azanowsky JM, Faliu B, Mansour Z, Chauvin F. Interaction between science advice and policymaking in time of COVID-19: a French perspective. Eur J Public Health. 2022;20:20.

4.	Easton M, Yarnold J, Vervaenen V, De Paepe J, Head BW. Expert perspectives on the changing dynamics of policy advisory systems: the COVID-19 crisis and policy learning in Belgium and Australia. POLICY AND POLITICS. 2024;52(1):67–87.

5.	Giozza SP, Bermudez XPD, Kara EO, Calvet GA, de Filippis AMB, Lacerda MVG, et al. An initiative of cooperation in Zika virus research: the experience of the ZIKABRA study in Brazil. BMC Public Health. 2021;21(1):572.

6.	Guleid FH, Njeru A, Kiptim J, Kamuya DM, Okiro E, Tsofa B, et al. Experience of Kenyan researchers and policy-makers with knowledge translation during COVID-19: a qualitative interview study. BMJ Open. 2022;12(6):e059501.

7.	Helmyati S, Dipo DP, Adiwibowo IR, Wigati M, Safika EL, Hariawan MH, et al. Monitoring continuity of maternal and child health services, Indonesia. Bull World Health Organ. 2022;100(2):144–54A.

8.	McSween-Cadieux E, Lane J, Hong QN, Houle A-A, Lauzier-Jobin F, Saint-Pierre Mousset E, et al. Production and use of rapid responses during the COVID-19 pandemic in Quebec (Canada): perspectives from evidence synthesis producers and decision makers. Health Research Policy and Systems. 2024;22(1):22.

9.	O'Donnell G, Begg H. Far from Well: The UK since COVID-19, and Learning to Follow the Science(s)*. Fisc Stud. 2020;41(4):761–804.

10.	Owek CJ, Guleid FH, Maluni J, Jepkosgei J, Were VO, Sim SY, et al. Lessons learned from COVID-19 modelling efforts for policy decision-making in lower- and middle-income countries. BMJ glob. 2024;9(11):e015247.

11.	Rozenblum SD. France’s multidimensional COVID-19 response. Ad Hoc Committees and the Sidelining of Public Health Agencies. In: Greer Scott L, King Elizabeth J, Fonseca EMd, Peralta-Santos A, editors. Coronavirus Politics. The Comparative Politics and Policy of COVID-19: University of Michigan Press; 2021. p. 264–79.

12.	Smith KE, Macintyre A, MacAulay M, Fafard P. Chief medical officers in the United Kingdom: maintaining ‘independence’ inside government. Journal of Public Health. 2024;47(1):54–61.

13.	Vickery J, Atkinson P, Lin L, Rubin O, Upshur R, Yeoh EK, et al. Challenges to evidence-informed decision-making in the context of pandemics: qualitative study of COVID-19 policy advisor perspectives. BMJ Glob Health. 2022;7(4).

14.	Waganew W, Azazh A, Worku A, Redae B, Sultan M, Walelegn M, et al. COVID 19 response in Ethiopia: Experiences of the National clinical advisory team. Ethiop J Health Dev. 2021;35(4):4.

15.	Weinkle J. An evaluation of North Carolina science advice on COVID-19 pandemic response. Hum Soc Sci Commun. 2022;9(1):352.

16.	Williamson L, McArthur E, Dolan H, Levesque JF, Sutherland K. Horizon scanning, rapid reviews and living evidence to support decision-making: lessons from the work of the Critical Intelligence Unit in New South Wales, Australia during the COVID-19 pandemic. BMJ Open. 2023;13(5):e071003.

17.	Yigzaw T, Kaba M, Mekonnen T, Mekonnen A, Molla M, Goji M, et al. Collaborative Engagement of Health Professional Associations in Ethiopia's COVID-19 Response: Achievements and Lessons Learned. Ethiop J Health Dev. 2021;35(4):4.

18.	Bastani P, Bahmaei J, Kharazinejad E, Samadbeik M, Liang Z, Schneider CH. How COVID-19 affects the use of evidence informed policymaking among iranian health policymakers and managers. Arch. 2022;80(1):16.

19.	Howard C, Hyland-Wood B. The promise and performance of data ecosystems: Australia's COVID-19 response. POLICY AND POLITICS. 2024;52(1):108–30.

20.	Aagaard P, Chatzopoulou S, Poulsen B. Analysing expert advice on political decisions in times of crisis. Policy & Politics. 2024;52(1):24–43.

21.	Abubakar I, Dalglish SL, Ihekweazu C, Bolu O, Aliyu SH. Lessons from co-production of evidence and policy in Nigeria's COVID-19 response. BMJ glob. 2021;6(3):03.

22.	Brusselaers N, Steadson D, Bjorklund K, Breland S, Sorensen JS, Ewing A, et al. Evaluation of science advice during the COVID-19 pandemic in Sweden. Hum Soc Sci Commun. 2022;9(1):17.

23.	Hadorn S, Sager F, Mavrot C, Malandrino A, Ege J. Evidence-Based Policymaking in Times of Acute Crisis: Comparing the Use of Scientific Knowledge in Germany, Switzerland, and Italy. Polit Vierteljahresschr. 2022:1–24.

24.	Hammersley M. Was the UK Government's Policymaking 'Evidence-Based' During the Pandemic? Reflections on Science and Politics. Zeitschrift für Evaluation. 2023;22(2):217–42.

25.	Hillmer MP, Feng P, McLaughlin JR, Murty VK, Sander B, Greenberg A, et al. Ontario's COVID-19 Modelling Consensus Table: mobilizing scientific expertise to support pandemic response. Can J Public Health. 2021;112(5):799–806.

26.	Jarman H, Falkenbach M, Rozenblum S, Rockwell O, Greer SL. From evidence to advice in France, Germany, and the UK: transparency, accountability, and participation in pandemic science advice. Journal of Public Health Policy. 2025;46(1):38–52.

27.	Karekla M, Hofer S, Plantade-Gipch A, Neto DD, Schjodt B, David D, et al. The Role of Psychologists in Healthcare During the COVID-19 Pandemic Lessons Learned and Recommendations for the Future. Eur J Psychol Open. 2021;80(1-2):5–17.

28.	Lopez Gomez A, Dogmanas D, Brunet-Adami N, Bagattini N, Bernardi R. Using behavioural and social sciences to inform public policies during COVID-19, Uruguay. Bull World Health Organ. 2021;99(11):843–4.

29.	Markovic-Denic L, Popadic D, Jovanovic T, Bonaci-Nikolic B, Samardzic J, Tomic Spiric V, et al. Developing COVID-19 vaccine recommendations during the pandemic: The experience of Serbia's Expert Committee on Immunization. Front Public Health. 2022;10:1056670.

30.	Matus K, Sharif N, Li A, Cai Z, Lee WH, Song M. From SARS to COVID-19: the role of experience and experts in Hong Kong's initial policy response to an emerging pandemic. Humanit Soc Sci Commun. 2023;10(1):9.

31.	McCabe R, Donnelly CA. Disease transmission and control modelling at the science-policy interface. Interface focus. 2021;11(6):20210013.

32.	Musa S, Jacques-Carroll L, Palo M. The role of National Immunization Technical Advisory Groups in advising COVID-19 immunization policy during the pandemic: lessons from the Federation of Bosnia and Herzegovina. Frontiers in Public Health. 2023;11.

33.	Pillai SK, Nyenswah T, Rouse E, Arwady MA, Forrester JD, Hunter JC, et al. Developing an incident management system to support Ebola response -- Liberia, July-August 2014. MMWR Morb Mortal Wkly Rep. 2014;63(41):930–3.

34.	Saito T, Muto K, Tanaka M, Okabe N, Oshitani H, Kamayachi S, et al. Proactive Engagement of the Expert Meeting in Managing the Early Phase of the COVID-19 Epidemic, Japan, February–June 2020. Emerging infectious diseases. 2021;27(10):1–9.

35.	Koppl R, Pronin K, Cowen N, Podemska-Mikluch M, Paniagua P. Bespoke science: the use of ad hoc scientific advisory committees in the Covid-19 pandemic. Public Choice. 2025;205(3):513–36.

36.	Eichenberger S, Varone F, Sciarini P, Stähli R, Proulx J. When do decision makers listen (less) to experts? The Swiss government's implementation of scientific advice during the COVID-19 crisis. Policy Studies Journal. 2023;51:587–605.

37.	Dattner I, Gal R, Goldberg Y, Goldshtein I, Huppert A, Kenett RS, et al. The role of statisticians in the response to COVID-19 in Israel: a holistic point of view. Isr J Health Policy Res. 2022;11(1):22.

38.	Gray A, Miot J, Mokgatle M, Rees H. 'Following the science': the role of an independent advisory structure in the COVID-19 pandemic response and beyond. South African Health Review. 2022;2022(1):1–10.

39.	König T, Stampfer M. "A Militia of Anarchists Run by a General". A Case of Scientific Policy Advice in Austria During the Pandemic. MINERVA. 2025.

40.	Vallejo BM, Ong RAC. OCTA as an independent science advice provider for COVID-19 in the Philippines. Hum Soc Sci Commun. 2022;9(1):9.

41.	Williams GA, Ulla Diez SM, Figueras J, Lessof S. Translating evidence into policy during the COVID-19 pandemic: bridging science and policy (and politics). (Special Issue: COVID-19 health system response.). Eurohealth. 2020;26(2):29–33.

42.	Marres N, Barragán MV. Making expert advice public in a time of emergency: Independent SAGE and the contestation of science during the Covid pandemic in the UK. Social Studies of Science. 2025.

43.	Pykett J, Ball S, Dingwall R, Lepenies R, Sommer T, Strassheim H, et al. Ethical moments and institutional expertise in UK Government COVID-19 pandemic policy responses: where, when and how is ethical advice sought? Evidence & Policy. 2023;19(2):236–55.

44.	Camporesi S, Angeli F, Fabbro GD. Mobilization of expert knowledge and advice for the management of the Covid-19 emergency in Italy in 2020. Humanities and Social Sciences Communications. 2022;9(1):1–14.

45.	Attwell K, Harper T, Carlson SJ, Tchilingirian J, Westphal D, Blyth CC. Facilitating knowledge transfer during Australia’s COVID-19 vaccine rollout: an examination of ‘Functional Dialogues’ as an approach to bridge the evidence–policy gap. Evidence & Policy. 2025;21(3):324–46.

46.	McGowran P, Johns H, Raju E, Ayeb-Karlsson S. The making of India's COVID-19 disaster: A Disaster Risk Management (DRM) Assemblage analysis. Int J Disaster Risk Reduct. 2023;93:103797.

47.	Capano G, Casula M, Malandrino A, Terlizzi A, Toth F. A new typology for comparing scientific advisory committees. Evidence from the Italian response to the COVID‐19 pandemic. Review of Policy Research. 2023.

48.	Wabnitz K, Rueb M, Rehfuess EA, Strahwald B, Pfadenhauer LM. Assessing the impact of an evidence- and consensus-based guideline for controlling SARS-CoV-2 transmission in German schools on decision-making processes: a multi-component qualitative analysis. Health Research Policy and Systems. 2023;21(1):138.

49.	Akbari-Sari A, Ahmadnezhad E, Abdi Z, Riazi-Isfahani S, Saffarpour S. Relevance of the COVID-19 rapid response system to public health policymaking in Islamic Republic of Iran. East Mediterr Health J. 2023;29(7):570–4.

50.	Colman E, Wanat M, Goossens H, Tonkin-Crine S, Anthierens S. Following the science? Views from scientists on government advisory boards during the COVID-19 pandemic: a qualitative interview study in five European countries. BMJ glob. 2021;6(9):09.

51.	Corrin T, Cairney P, Kennedy EB. The production and utility of evidence synthesis during the COVID-19 pandemic in Canada: perspectives of evidence synthesis producers. EVIDENCE & POLICY. 2025;21(1):108–28.

52.	Georgalakis J. A disconnected policy network: The UK's response to the Sierra Leone Ebola epidemic. Social Science & Medicine. 2020;250:112851.

53.	de Andrade KRC, Carvalho VKD, Silva RB, Luquine CD, Jr., Farinasso CM, Oliveira CD, et al. Evidence syntheses to support decision-making related to the Covid-19 pandemic. REVISTA DE SAUDE PUBLICA. 2024;58.

54.	Dobbins M, Dubois A, Atkinson D, Bellefleur O, Betker C, Haworth-Brockman M, et al. Nimble, efficient and evolving: the rapid response of the National Collaborating Centres to COVID-19 in Canada. Health Promot Chronic Dis Prev Can. 2021;41(5):165–70.

55.	El-Jardali F, Bou-Karroum L, Fadlallah R. Amplifying the role of knowledge translation platforms in the COVID-19 pandemic response. Health Res Policy Syst. 2020;18(1):58.

56.	Neil-Sztramko SE, Belita E, Traynor RL, Clark E, Hagerman L, Dobbins M. Methods to support evidence-informed decision-making in the midst of COVID-19: creation and evolution of a rapid review service from the National Collaborating Centre for Methods and Tools. BMC Medical Research Methodology. 2021;21(1):231.

57.	Rankin NM, Nutbeam D, Levesque J-F, Ko H, Jennings G, Walczak A, et al. COVID-19 research response to immediate demands: setting priorities with key stakeholders to enable health services research in NSW, Australia. Journal of Health Organization and Management. 2024;38(9):344–59.

58.	Christensen T, Lægreid P. Scientization Under Pressure—The Problematic Role of Expert Bodies During the Handling of the COVID-19 Pandemic. Public Organization Review. 2022;22:291–307.

59.	Hasler B, Bazeyo W, Byrne AW, Hernandez-Jover M, More SJ, Ruegg SR, et al. Reflecting on One Health in Action During the COVID-19 Response. Front. 2020;7:578649.

60.	Groot G, Witham S, Badea A, Baer S, Dalidowicz M, Reeder B, et al. Evaluating a learning health system initiative: Lessons learned during COVID-19 in Saskatchewan, Canada. Learn Health Syst. 2022;7(3):e10350.
